# The primary vascular dysregulation syndrome: implications for eye diseases

**DOI:** 10.1186/1878-5085-4-14

**Published:** 2013-06-07

**Authors:** Josef Flammer, Katarzyna Konieczka, Andreas J Flammer

**Affiliations:** 1 Department of Ophthalmology, University of Basel, Mittlere Strasse 91, Basel CH-4031, Switzerland; 2 Cardiovascular Center, Cardiology, University Hospital Zurich, Zurich CH-8091, Switzerland

**Keywords:** Primary vascular dysregulation, Endothelial dysfunction, Vasospasm, Glaucoma, Retinal venous pressure, Risk factors, Molecular targets, Predictive diagnostics, Targeted prevention, Integrative medical approach

## Abstract

Vascular dysregulation refers to the regulation of blood flow that is not adapted to the needs of the respective tissue. We distinguish primary vascular dysregulation (PVD, formerly called vasospastic syndrome) and secondary vascular dysregulation (SVD). Subjects with PVD tend to have cold extremities, low blood pressure, reduced feeling of thirst, altered drug sensitivity, increased pain sensitivity, prolonged sleep onset time, altered gene expression in the lymphocytes, signs of oxidative stress, slightly increased endothelin-1 plasma level, low body mass index and often diffuse and fluctuating visual field defects. Coldness, emotional or mechanical stress and starving can provoke symptoms. Virtually all organs, particularly the eye, can be involved. In subjects with PVD, retinal vessels are stiffer and more irregular, and both neurovascular coupling and autoregulation capacity are reduced while retinal venous pressure is often increased. Subjects with PVD have increased risk for normal-tension glaucoma, optic nerve compartment syndrome, central serous choroidopathy, Susac syndrome, retinal artery and vein occlusions and anterior ischaemic neuropathy without atherosclerosis. Further characteristics are their weaker blood–brain and blood-retinal barriers and the higher prevalence of optic disc haemorrhages and activated astrocytes. Subjects with PVD tend to suffer more often from tinnitus, muscle cramps, migraine with aura and silent myocardial ischaemic and are at greater risk for altitude sickness. While the main cause of vascular dysregulation is vascular endotheliopathy, dysfunction of the autonomic nervous system is also involved. In contrast, SVD occurs in the context of other diseases such as multiple sclerosis, retrobulbar neuritis, rheumatoid arthritis, fibromyalgia and giant cell arteritis. Taking into consideration the high prevalence of PVD in the population and potentially linked pathologies, in the current article, the authors provide recommendations on how to effectively promote the field in order to create innovative diagnostic tools to predict the pathology and develop more efficient treatment approaches tailored to the person.

## Table of contents

1. Introduction

2. Regulation of blood flow

2.1. Function of blood flow

2.1. Regulation of blood flow in general

2.2. Global blood circulation

2.2. Blood supply to the individual organs

2.2. The role of vascular endothelial cells

2.1. Regulation of ocular blood flow

2.2. Anatomy of ocular blood supply

2.2. Regulation of retinal blood flow

2.2. Regulation of choroidal blood flow

2.2. Regulation of optic nerve head blood flow

3. Vascular dysregulation

3.1. Primary vascular dysregulation (PVD)

3.1.1. Risk factors for PVD

3.1.1. Pathomechanisms of PVD

3.1.1. Trigger factors for PVD

3.1.1. PVD and Raynaud’s disease

3.1. Secondary vascular dysregulation

3.1.1. Mechanism of SVD

3.1.1. Causes of SVD

4. General symptoms and signs of PVD

4.1. General symptoms of PVD

4.1.1. PVD and temperature sensation

4.1.1. PVD and thirst

4.1.1. PVD and sleep behaviour

4.1.1. PVD and physical activity

4.1.1. PVD and professional activity

4.1.1. PVD and sensitivity

4.1.1. PVD and psychological stress

4.1.1. PVD and pain

4.1.1. PVD and migraine

4.1.1. PVD and altitude sickness

4.1.1. PVD, tinnitus and sudden hearing loss

4.1.1. PVD and thyroid dysfunction

4.1. General signs of PVD

4.1.1. PVD and temperature

4.1.1. PVD and Endothelin-1

4.1.1. PVD and blood pressure

4.1.1. PVD and the heart

4.1.1. PVD and the autonomic nervous system

4.1.1. PVD and circadian rhythm

4.1.1. PVD and gene expression in lymphocytes

4.1.1. PVD and oxidative stress

5. Visual signs and symptoms of PVD

5.1. PVD and visual function

5.1. PVD and ocular blood flow

5.1. PVD and retinal vascular spatial irregularities

5.1. PVD and stiffness of retinal vessels

5.1. PVD and neurovascular coupling

5.1. PVD and blood–brain barrier

5.1. PVD and activation of astrocytes

5.1. PVD and autoregulation

5.1. PVD and the relation between peripheral and ocular circulation

5.1. PVD and retinal venous pressure

5.1. PVD and optic disc haemorrhages

6. PVD and ophthalmic diseases

6.1. PVD and glaucoma

6.1. PVD and other eye diseases

6.2. Retinal arterial occlusion

6.2. Retinal vein occlusion

6.2. Anterior ischaemic optic neuropathy

6.2. Susac syndrome

6.2. PVD and optic nerve compartment syndrome

6.2. Central serous chorioretinopathy

6.2. Leber hereditary optic neuropathy

6.2. Retinitis pigmentosa

7. Therapy

7.1. Lifestyle

7.1. Nutrition

7.1. Magnesium

7.1. Calcium channel blockers

7.1. Endothelin-antagonists

7.1. Fludrocortisone

7.1. Carboanhydrase inhibitors

7.1. Ginkgo biloba

7.1. Other drugs

8. Conclusion

## 1. Introduction

The amount of blood needed in various organs and tissues varies greatly over time, which is why there is a demand for a sophisticated blood flow (BF) regulation. This is achieved by adapting perfusion pressure and local resistance to flow; resistance, thereby, is a function of the vessel diameter. The term *vasospasm* denotes an inappropriate constriction of an artery [1]. If such spasms occur in several organs simultaneously or sequentially, the condition is referred to as vasospastic syndrome [2]. The term *vascular dysregulation*, however, is a broader term that embraces arterial spasms and also inappropriate constrictions and/or dilatation of arteries, veins or capillaries, often accompanied by a barrier dysfunction [3,4]. Vascular dysregulation can lead to both over and under perfusion of a particular supply territory. If vascular dysregulation is associated with symptoms or signs, the term *vascular dysregulation syndrome* is used. This syndrome can be *primary* (primary vascular dysregulation syndrome, PVD syndrome) or *secondary* to another disease (secondary vascular dysregulation syndrome, SVD syndrome). Vascular dysregulation can involve any organ; however, the eye is particularly often affected. In this review, we summarise the basic aspects of vascular dysregulation and focus on its role in the pathogenesis of eye diseases.

## 2 Regulation of blood flow

### 2.1 Function of blood flow

One of the major functions of BF is its transport capability. It transports not only cells, ions and molecules but, importantly, also heat. In a nutshell, blood circulation connects organs, allowing the body to function as an integrative system.

Adequate and inadequate BF has a huge impact on human health and disease. The generally best known causes of disturbed BF are related to the consequences of advanced atherosclerosis and/or its secondary events like thrombosis and embolism. The risk factors for atherosclerosis have been studied extensively. Its medical and socio-economic impacts are most visible after a cerebrovascular insult or myocardial infarction. Vascular dysregulation is another, but far less well known, cause of a disturbed BF to organs, and its often-neglected role in the pathogenesis of several diseases will be discussed in detail in this review.

### 2.2 Regulation of blood flow in general

There are two different regulatory components: the global circulation of the entire body and the distribution of BF to the individual organs or parts of them.

#### 2.2.1 Global blood circulation

The heart pumps blood into the arteries, building up a pressure gradient from the arteries over the capillaries to the veins back to the heart. The global circulation equals the cardiac output, which is the amount of blood that is pumped by the heart per unit of time. This cardiac output is a function of heart rate and stroke volume. It is constantly adapted to the overall need of the body. During physical activity, for example, it can be four to five times larger than when at rest. Cardiac output is mainly controlled by the autonomic nervous system and circulating hormones.

Blood pressure results from the relationship between cardiac output and the overall resistance to flow. Blood pressure in itself, therefore, does not reflect the overall BF or the BF to the individual organs. In other words, a patient with systemic arterial hypertension or hypotension does not automatically have a higher or a lower overall perfusion.

#### 2.2.2 Blood supply to individual organs

The demand of BF to different organs or parts of the organs varies partially independently. The distribution of cardiac output is therefore regulated by adapting the relative local resistance.

The BF to a tissue depends on the relationship between local perfusion pressure and local resistance to flow. Therefore, perfusion pressure alone is a weak parameter for the BF of an organ with autoregulation. If, however, the local regulation of BF in an organ is disturbed or absent, perfusion pressure becomes the dominant factor. Vascular resistance is regulated particularly in arteries and, to some extent, also in capillaries and veins. An increased resistance in the veins increases venous pressure and thereby reduces perfusion pressure and also increases transmural pressure in the capillaries. This leads potentially to higher extravasation of fluid.

The size of the vessels is regulated by contraction (vasoconstriction) or relaxation (vasodilatation) of the smooth muscle cells in arteries and veins and of pericytes in the capillaries. The smooth muscle cells get the corresponding information from different sources. They sense tension (myogenic regulation), and a large number of factors such as K^+^, H^+^ or CO_2_ originating from the surrounding tissues influence smooth muscle cells either directly or indirectly via endothelial cells. In addition, smooth muscle cells get information from the autonomic nervous system and from molecules circulating in the blood.

#### 2.2.3 The role of vascular endothelial cells

**Figure 1:**
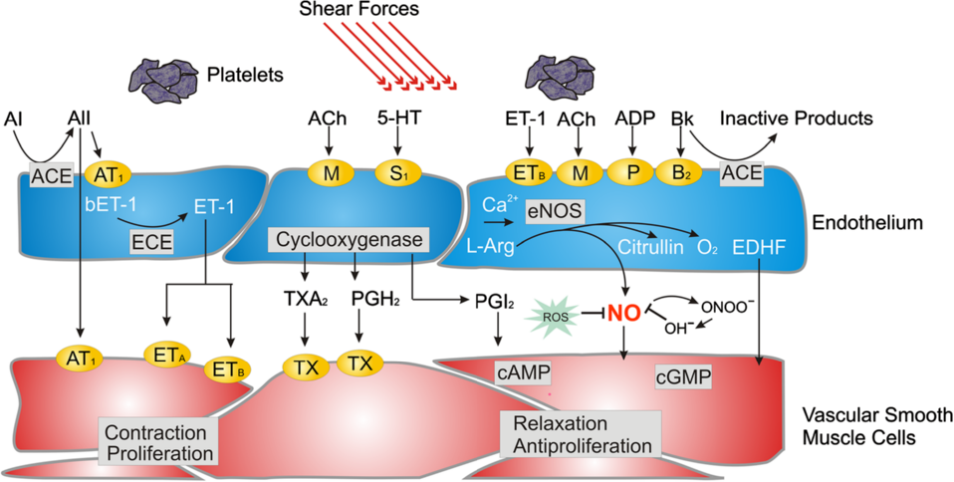
**VEC receive physical and chemical information, integrate it and release vasoactive substances regulating vascular tone.** (From [5], with permission).

The endothelium is a thin layer of cells lining the inner parts of arteries, capillaries, veins and lymphatic vessels, thereby forming a structural barrier between the vascular space and the surrounding tissue. It is a dynamic organ, important in several housekeeping functions such as haemostasis, barrier function, immune and inflammatory responses, angiogenesis and particularly in the regulation of vascular tone. It has autocrine, paracrine and endocrine functions (Figure 1). It perceives mechanical information like sheer stress and traction as well as biochemical information, particularly via the circulating molecules [5-7]. Multiple factors, such as cardiovascular risk factors, genetic factors and inflammation, can influence the endothelium and predispose a subject to endothelial dysfunction [8]. A dysfunctional endothelium is involved in the pathogenesis of various diseases, which are called *endotheliopathies*[9]. A dysfunction of vascular endothelial cells (VEC) also plays a role in eye diseases. There is, for example, a relationship between reduced vascular endothelial progenitor cells and reduced flow-mediated dilation in glaucoma patients [10]. We also gain insight into the function of VEC from the analysis on neurovascular coupling in the retina. Stimulation with a flickering light leads to a quick dilatation of small vessels and, thereby, to a dilatation of larger retinal vessels by a mechanism called flow-mediated vasodilatation. A prerequisite for this dilatation is a normal VEC function. Interestingly, reduced or even absent response, as quantified by the retinal vessel analyser, can be observed in both healthy PVD subjects [11] and in glaucoma patients progressing despite an intraocular pressure (IOP) in the normal range [12].

### 2.3 Regulation of ocular blood flow

#### 2.3.1 Anatomy of ocular blood supply

The eye is supplied by two vascular systems: the uveal and the retinal vessels. The uveal vascular system supplies the iris, the ciliary body and the choroid and also, by diffusion, the outer layers of the retina, including the photoreceptors. The retinal vessels supply the inner layers of the retina including the retinal ganglion cells.

Both the uveal and retinal vessels originate from the ophthalmic artery, which in turn is a branch of the internal carotid artery. The central retinal artery provides blood, and the central retinal vein drains the retina. The anterior choroid is supplied by the long ciliary arteries, whereas the posterior choroid is supplied by the short posterior ciliary arteries. The entire choroid drains into the vortex veins.

The retinal BF is characterised by a low perfusion rate, a high vascular resistance and a high oxygen extraction. By contrast, the choroid shows a high perfusion rate, a low vascular resistance and a low oxygen extraction.

The BF in different tissues in the eye is regulated differently [13]. These regulations will therefore be discussed separately.

#### 2.3.2 Regulation of retinal blood flow

Retinal vessels are very similar to brain vessels. However, only the extraocular part but not the intraocular part has autonomic innervation [14]. Proper retinal function requires the presence of a blood-retinal barrier. This physiological barrier regulates the flux of ions, proteins, hormones and water and also regulates infiltration of immune competent cells.

Retinal BF depends on the relationship between perfusion pressure and local resistance. Perfusion pressure is the difference between retinal arterial and retinal venous pressure. Under physiological conditions, retinal venous pressure is often more or less equal to the IOP. There are, however, a number of conditions in which retinal venous pressure is distinctly higher than the IOP [15].

Retinal BF, similar to brain BF, is autoregulated. Autoregulation is the intrinsic capacity to maintain constant flow despite changes in perfusion pressure. Autoregulation, however, is effective only within certain limits of perfusion pressure.

The size of retinal vessels and, thereby, the retinal circulation are also influenced by retinal activity. This connection is called neurovascular coupling. Neuronal activity in the central nervous system (including the retina) evokes localised changes in BF. The magnitude and the spatial location of these changes are tightly linked to changes in neural activity through a complex sequence of coordinated events involving neurons, glia and the vascular walls. The neurovascular coupling also provides a basis for functional imaging. If flickering light hits the retina, blood vessels dilate within seconds. The direct visual accessibility of fundus blood vessels allows direct monitoring of this response *in vivo*.

#### 2.3.3 Regulation of choroidal blood flow

**Figure 2:**
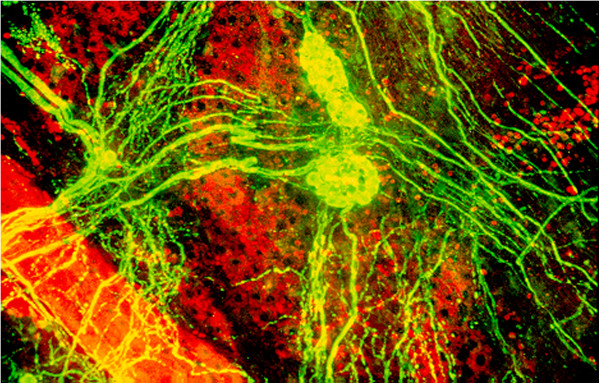
**Confocal microscopy of a whole mount from a Rhesus monkey choroid.** After staining of neural structures with a neuronal marker. Groups of ganglion cells are connected by axon bundles. Choroidal arteries are densely surrounded by perivascular nerve fibres. (From [20], with permission).

Choroidal BF regulation is distinctly different from the regulation of retinal BF [16,17]. The choroidal vessels are extensively innervated [18,19] (Figure 2). In addition to providing oxygen and other molecules, choroidal circulation also regulates eye temperature and most likely contributes to the fine-tuning of accommodation by regulating choroid thickness. This vascular bed is only partly autoregulated. The capillaries are fenestrated. Therefore, there is no or only little oncotic pressure gradient between intra- and extravascular spaces. The lack of oncotic pressure together with the absence of lymphatic vessels explains the choroidal effusion if IOP drops below a certain level.

Ciliary BF is similar to choroidal BF. Blood supply to the ciliary body is a prerequisite for the formation of aqueous humour [21]. A major drop of ciliary body BF therefore leads to a reduction of aqueous humour formation and, thereby, to a reduction of IOP.

#### 2.3.4 Regulation of optic nerve head blood flow

**Figure 3:**
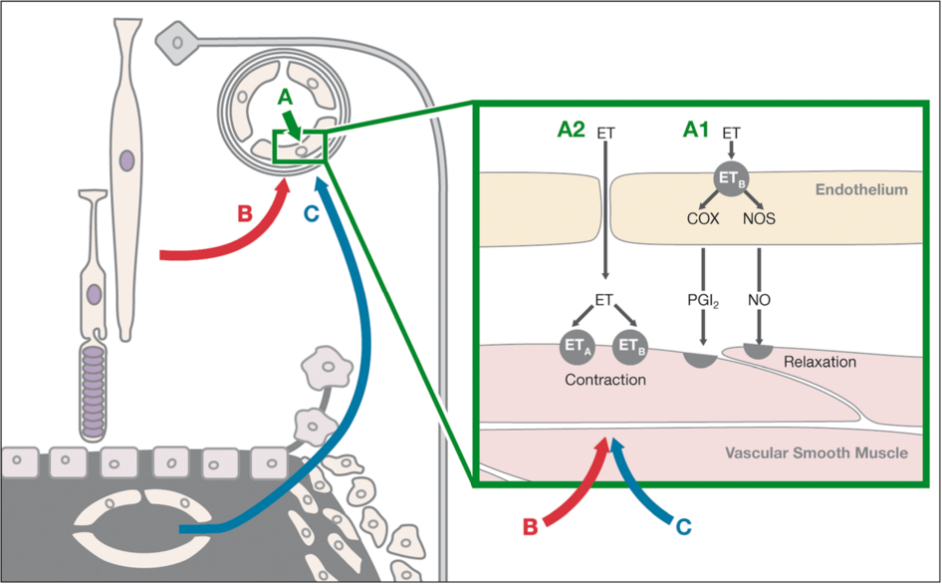
**Influence of ET-1 on blood vessels in the optic nerve head and adjacent retina.** (**A**) Circulating ET-1 reaches the endothelial cells (**A1**) and has a more or less neutral effect. If the blood–brain barrier is disrupted, it reaches directly the vascular smooth muscle cells (**A2**) and induces vasoconstriction. (**B**) Hypoxic retina produces ET-1 diffusing to neighbouring vessels. (**C**) ET-1 diffuses from fenestrated capillaries into the optic nerve head and adjacent retina, leading to vasoconstriction and, thereby, also to increased retinal venous pressure. (Modified from [26], with permission).

Blood supply to the optic nerve head (ONH) is very unique by any standard. The superficial layer of the ONH receives its blood supply via small branches of the central retinal artery. The prelaminar region (a small area anterior to the lamina cribrosa), however, is mainly supplied by branches from the choroidal arteries and directly from the short posterior ciliary arteries. The prelaminar area contains no arterioles, but has capillaries that are exceptionally long. In contrast to the arterial supply, venous drainage of the ONH is through the central retinal vein. The autoregulation in this area is less efficient than in the retina but better than in the choroid [22,23]. The ONH seems to be the only part of the central nervous system that has no proper blood–brain barrier. This is due to a lack of a proper blood–brain barrier of its capillaries [24] and to the fact that molecules can diffuse into the ONH from the surrounding choroid [25]. The diffusing vasoactive substances influence both size and barrier function of the vessels in the ONH and the adjacent retina. This is exemplified by endothelin-1 (ET-1) in Figure 3. Increased plasma concentrations of ET-1 reach the vascular endothelial cells and exert a more or less neutral effect on the vessel size, as long as the blood–brain barrier is intact. If, however, the blood–brain barrier is disrupted, ET-1 gets direct access to the vascular smooth muscle cells. If the proximate retina is hypoxic, hypoxia-inducible factor-1α (HIF-1α) is increased and acts as a transcription factor, stimulating (among other molecules) the production of vascular endothelial growth factor (VEGF) or ET-1. This leads to both vasoconstriction and weakening of the blood–brain barrier in the corresponding area. Circulating ET-1 can also diffuse from the fenestrated choriocapillaries directly into the ONH and adjacent retina bypassing the blood–brain barrier. Therefore, both local pathologies and systemic changes have a potential impact on ONH vessels, both arteries and veins (Figure 3).

## 3 Vascular dysregulation

The need for BF to different organs varies, and this is adapted by local vascular regulation. Such an adaption can be insufficient or improper. A patient with intermittent claudication, for example, cannot - due to atherosclerosis - properly increase blood supply to the muscles of the legs while walking. The adaption of BF, however, can also be insufficient, despite anatomically normal vessels, due to a dysfunctional regulation of vascular tone [1,4]. This may be illustrated by red and white blotches on the face and neck of certain people when they are stressed. This is harmless but illustrates the basic characteristics of vascular dysregulation: the blood supply to a given organ is, for a period of time, not properly adapted to the need of this organ. Vascular dysregulation stands for an inadequate constriction or insufficient dilatation, as well as excessive dilatation of arteries, capillaries or veins. Such vascular dysregulation is often associated with other vascular dysfunctions, such as weakening of the blood–brain barrier [26] and also with seemingly independent signs and symptoms [27].

Global vascular dysregulation syndromes can be divided into primary vascular dysregulation and secondary vascular dysregulation syndromes. PVD is a predisposition to respond differently to a variety of stimuli like coldness or emotional stress. SVD is a dysfunction of anatomically healthy vessels, induced secondarily by an underlying disease [28].

### 3.1 Primary vascular dysregulation

Because PVD is an important risk factor for several eye diseases [1,3], we will discuss this entity in more detail. As outlined above, the PVD syndrome is a general predisposition to respond differently than unaffected persons do to a variety of stressors and stimuli, such as coldness, emotional stress and others. The most prominent aspect is the dysregulation of the vascular tone, giving the syndrome its name. However, other clinical signs and symptoms are often associated as well. Due to the lack of a gold standard for diagnosis and differences among populations, we do not know the exact prevalence of PVD. While in Switzerland 31% of women and 7% of men complain of cold extremities [29], only about 10% of women and 3% of men show the classic symptoms of PVD [30]. The term PVD should not be confused with the term *posterior vitreous detachment*.

#### 3.1.1 Risk factors for PVD

Most of the subjects with PVD indicate that a parent suffered from the same syndrome. It is therefore likely an inherited disposition.

The syndrome manifests itself at, or shortly after, puberty and mitigates or even disappears with older age, particularly in females after menopause. The prevalence is much higher in women than in men [29]. Like migraine [31], PVD symptoms can recur if females take oestrogen replacement therapy after menopause. Therefore, oestrogen most likely plays a role as well.

Based on clinical experience, the syndrome occurs more often in academics than in blue-collar workers and more in Asians than in Caucasians. Although all these associations are obvious, the causal relationship is still unclear. The gender difference may partly be explained by differences in distribution of adrenergic receptors [32]. PVD is much more prevalent in slim people than in obese people [33]. Interestingly, patients with normal-tension glaucoma (NTG) also tend to be slim [34], and progression of NTG is often faster in patients with low body mass index (BMI) [35,36].

#### 3.1.2 Pathomechanisms of PVD

The exact pathogenesis for the development of PVD is still unclear. On the one hand, the autonomic nervous system is involved and contributes to the symptoms [37,38], but on the other hand, the un-innervated retinal vessels are also involved. Therefore, PVD cannot be explained by the dysfunction of the autonomic nervous system only. Importantly, endothelial dysfunction is also involved; however, it is unclear what mechanism leads to such a type of endotheliopathy. An imbalance of ET-1 and nitric oxide (NO) production is most likely a major player and has been observed in glaucoma patients [39]. The mitochondria are also involved, although it is difficult to know whether they play a primary role or if they are involved secondarily [40,41].

#### 3.1.3 Trigger factors for PVD

Subjects with a predisposition for PVD have only minor symptoms, as long as they are not challenged. A classical challenge is coldness [2,42]. Subjects report more often the feeling of freezing, and they exhibit cooler extremities [43] and even cooler corneas than others when exposed to coldness [44]. Another trigger factor is psychological stress [45]. The way our body responds to this stress seems to be individually different [46] and also gender dependent [47]. Subjects with PVD often respond with redistribution of BF. These persons may get cold hands during stressful periods. To give an example, we performed a nailfold capillary microscopy test on a female musician suffering from NTG, a condition associated with PVD. While baseline BF in the capillaries was normal and her cold-induced BF stop lasted only a few seconds, the BF in the nailfold capillaries stopped for about 3 min when she started to talk about problems with her husband. Additionally, we observed a more heterogeneous distribution of temperature during psychological stress in thermography of the face as well.

In addition to cold and emotional stress, there are many other trigger factors. While some PVD subjects respond more to one particular trigger, others respond to multiple triggers. Vibration is another stimulus leading to vasoconstriction in some people [48], interestingly not only in the exposed but also in the unexposed hand [49]. Such people cannot work with a compressor [50]. Workers with hand-arm vibration syndrome also suffer from cold-induced vasospasm [51], indicating an underlying predisposition. Rarely do subjects with PVD respond to physical exercise. We made the observation that PVD subjects tend to suffer longer and stronger from whiplash traumata than others. Indeed, reduced cold pain threshold (cold hyperalgesia) predicted a worse outcome after whiplash traumata [52,53].

#### 3.1.4 PVD and Raynaud's disease

**Figure 4:**
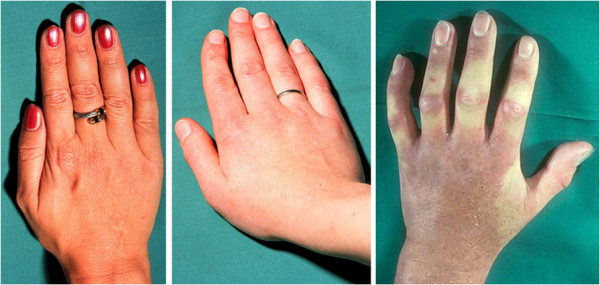
**Raynaud's disease versus PVD.** (Left) Hand of a healthy person. (Middle) Hand of a PVD subject. (Right) Hand of a patient with Raynaud's disease. (From [57], with permission).

PVD [1] and Raynaud's disease [54] have certain similarities, which is why they are often confused with each other. In his thesis, written in 1892, Raynaud described an illness involving local ischaemic of the hands. This led to the term *Raynaud's disease*. In Raynaud's disease, the fingers turn white, which is why it is sometimes called the ‘white finger disease’ or ‘corpse finger disease’ (Figure 4). In addition, Raynaud's disease leads to morphological changes in the fingertips. Raynaud's disease is therefore distinctly different from PVD. Later, the term *Raynaud's phenomenon* was coined to describe a similar but clearly weaker entity than Raynaud's disease. Raynaud's phenomenon can also be primary or secondary [55]. The symptoms of Raynaud's phenomenon are to some extent similar to those of PVD [54]. The hands of PVD subjects indeed are also cool, but in contrast to Raynaud's syndrome, they almost never turn totally white and are not numb. In addition, the onset of symptoms is not so abrupt and lasts longer than in Raynaud's phenomenon. PVD patients also suffer from a variety of other symptoms which do not occur in Raynaud's phenomenon. Therefore, PVD syndrome should not be confused with Raynaud's disease or with Raynaud's phenomenon. Additionally, both Raynaud's disease and Raynaud's phenomenon, in contrast to PVD, are only weakly related to glaucoma. Furthermore, PVD is also not identical with the so-called purple digit syndrome [56].

### 3.2 Secondary vascular dysregulation

BF to an organ can be dysregulated in diseases affecting the corresponding organ and also in diseases affecting remote organs. If a vascular dysregulation is secondary to such a disease, it is called secondary vascular dysregulation [1]. SVD in this context should not be confused with *small vessel diseases*.

#### 3.2.1 Mechanism of SVD

In various conditions, particularly in inflammatory diseases, there is a release of molecules into the corresponding tissue and partially also into the blood stream, leading to changes of molecule concentration in the circulating blood that affects remote organs. Well known is the release of tumour necrosis factor-alpha which, among many effects, influences temperature control in the hypothalamus, thereby inducing fever. In a similar way, other molecules influence vascular tone; among these molecules, the most important is ET-1. ET-1 is produced by the vascular endothelium cells and is released predominantly abluminally and only partially intraluminally [58]. Under stress conditions, however, other cells also start to produce ET-1 [59] and thereby influence its concentration in the circulating blood. An increase in ET-1 has little or no effect on brain or retinal circulation as long as the blood–brain or blood-retinal barrier is intact [60]. In case of blood–brain or blood-retinal barrier breakdown, ET-1 gets direct access to smooth muscle cells or pericytes, thereby leading to vasoconstriction. As no such barrier exists in the choroid, an increase in ET-1 reduces choroidal BF. There is also a ‘physiological barrier defect’ in the ONH because molecules can diffuse from the choroid into the ONH [25] (see Figure 3). This explains ONH BF reduction in all conditions with increased ET-1 plasma levels.

#### 3.2.2 Causes of SVD

We will discuss a few diseases which can lead to increased ET-1 plasma levels.

**Figure 5:**
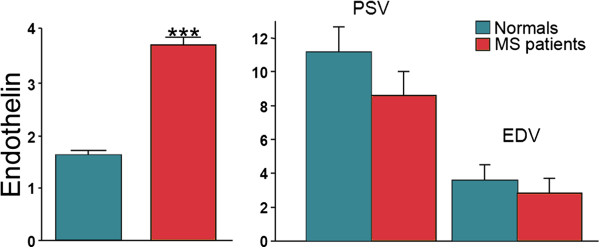
**Endothelin-1 in multiple sclerosis patients.** In some patients with multiple sclerosis, the plasma level of ET-1 (mean ± SEM) is increased (left; modified from [62]). This leads to a decrease of OBF. Peak systolic and end diastolic velocity (cm/s) in the medial posterior ciliary arteries (mean ± SEM) in the corresponding MS patients (right; from [69], with permission).

*Multiple sclerosis*. In patients with multiple sclerosis (MS), monocytes produce ET-1, thereby increasing its concentration in the cerebrospinal fluid (CSF). ET-1 levels are significantly elevated in the CSF of relapsing remitting MS patients with an acute clinical attack in comparison with those in a stable phase [61]. In some MS patients, ET-1 in the circulating blood [62] is increased, and therefore, ocular blood flow (OBF) decreased (Figure 5). This might contribute to the loss of retinal ganglion cells and their axons [63], to subclinical visual field defects [64], to narrower retinal arterioles and wider venules [65] and to increased rigidity of the retinal vessels [66]; furthermore, it may partially be the reason the ONH can turn slightly pale. Red wine can reduce ET-1 concentration which therefore explains why certain symptoms like visual disturbances in MS patients are reduced by red wine for a limited period of time [67]. The observed breakdown of the blood–brain barrier in these patients might also partly be due to ET-1 [68].

*Optic neuritis*. During optic neuritis, the blood ET-1 level rises quickly and then drops slowly during the next weeks but often remains slightly above normal values [70]. The reason for this increase during optic neuritis is not clear. Most likely, it is due to an increased production of ET-1 by monocytes. Whether ET-1 contributes to the symptoms is not clear at present.

*Rheumatoid arthritis*. Patients with rheumatoid arthritis have moderately increased plasma levels of ET-1 [71] produced, partly, by activated synovial cells [72]. ET-1 lowers the threshold of pain sensation. The mechanical hyperalgesia lasts longer in females [73], and ET-1 receptor blockade reduces pain [74].

*Fibromyalgia*. Some patients with fibromyalgia have increased levels of ET-1, although we do not know why [75]. It is also not known whether this plays any role. Nevertheless, by influencing the pain sensation threshold, the increased level of ET-1 presumably contributes to the pain sensation of these patients.

*Giant cell arteritis*. Giant cell arteritis is a systemic vasculitis affecting large and medium-sized arteries. The arteritic lesions lead to ischaemic symptoms such as cephalgia, masseter pain and mental depression. The acute loss of visual function is most often due to anterior ischaemic optic neuropathy and less frequently due to a central artery occlusion. Giant cell arteritis patients with involvement of the visual system have increased ET-1 plasma levels [76,77]. In inflamed temporal arteries, not only is the ET-1 level increased but also the expression of ET receptor B is upregulated both in vascular smooth muscle cells and multinucleated giant cells [78]. The involvement of the endothelin system explains why BF is reduced to a greater extent in the choroid than in the retina [79], why infarctions occur more often in the ONH than in the retina and also why amaurosis fugax-like symptoms often precede the major event [80]. The increased levels of ET-1 may be partly due to the matrix metalloproteinases (MMP2) released during inflammation. MMP2 converts the precursor molecule Big ET-1 to the active ET-1.

**Figure 6:**
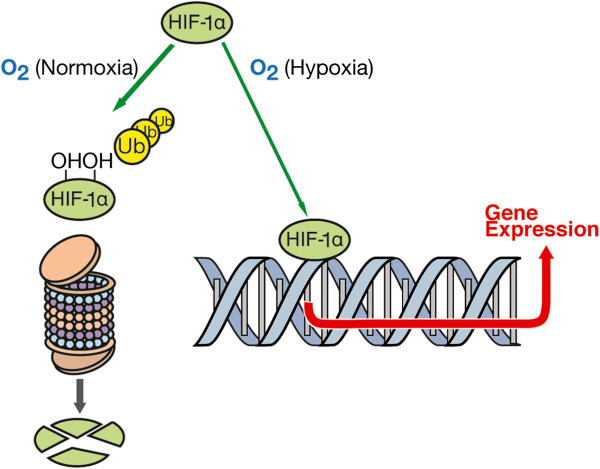
**Hypoxia-inducible factor-1α.** If oxygen concentration in a cell is lowered, less HIF-1α is oxidised and degraded. Thus, stabilised HIF-1α moves into the nucleus of the cell, where it acts as a transcription factor, stimulating the production of ET-1 or VEGF. (From [83], with permission.)

*Retinal vein occlusion*. Most patients with retinal vein occlusions have increased plasma levels of ET-1 during the acute phase. Weeks or months later, the concentration decreases, but rarely normalises totally [81,82]. The cause is unclear. The fact that the majority of patients suffer from other diseases such as systemic hypertension, diabetes or glaucoma makes it probable that these diseases may have caused or at least contributed to the increase of ET-1 plasma levels. PVD is another cause. For this reason, vein occlusions will be discussed in the section on PVD. The occlusion of the retinal vein seems to be often a secondary consequence of a retinal hypoperfusion due to an arterial disease. This leads to tissue hypoxia and thereby to an increase in HIF-1α (Figure 6).

HIF-1α then increases the expression of several factors like VEGF, erythropoietin and ET-1. An increased concentration of ET-1, both in the circulating blood and locally in the retinal tissue, can induce venous constriction and thereby increase retinal venous pressure [84]. If this pressure increases further, a pre-stasis syndrome and eventually the clinical picture of retinal vein occlusion (also called retinal venous thrombosis) develop. Indeed, ET-1 injection into the vitreous occludes the veins [85].

*Others*. Secondary vascular dysregulation may occur also in patients with antiphospholipid syndrome, pre-eclampsia, Susac syndrome and central serous chorioretinopathy. However, based on their medical history, these subjects also often suffer from PVD syndrome before they get sick; therefore, some of these diseases will be discussed in the context of PVD.

## 4 General symptoms and signs of PVD

Although the most striking signs and symptoms in PVD subjects are directly related to the dysregulation of BF, these subjects often have additional signs and symptoms only indirectly related or seemingly even unrelated to BF. These signs and symptoms are not specific for PVD subjects but occur significantly more often in PVD subjects than in non-PVD subjects. The knowledge on these signs and symptoms helps in understanding these subjects better and might influence recommendations for behaviour as well as treatment of such patients.

### 4.1 General symptoms of PVD

#### 4.1.1 PVD and temperature sensation

Classically, PVD patients indicate that they often feel cold, particularly in the hands or feet. Cold hands can be provoked not only by coldness but also by emotional stress, by vibration or via vasoconstrictive drugs; interestingly, the sensitivities to these trigger factors are interrelated. For example, emotional stress increases drug sensitivity, and patients that respond to vibration also have higher probability to respond to coldness [51,86,87]. An epidemiological study analysed thermal discomfort with cold extremities in a Swiss urban population. Younger subjects suffered more intensively from cold extremities than elderly subjects, women suffered more often than men, and slim subjects suffered more often than those with higher body mass index [33]. Psychological stress leads to vascular dysfunction, even in animals [88,89]. Subjects with PVD, however, seem to respond stronger to psychological stress than non-PVD subjects. There is also a relationship between cold extremities and sleep behaviour, which will be discussed in the section 4.1.3.

#### 4.1.2 PVD and thirst

The desire to drink is significantly lower in subjects with PVD, while estimated daily fluid intake is only slightly lower [27]. However, PVD subjects tend to forget to drink. The concentration of ET-1 in the circulating blood is slightly increased [27], and this (among other effects) suppresses - via the prostaglandin-E2 axes - the centre of thirst in the brain. Reduced feeling of thirst, however, occurs also in patients with other diseases in which ET-1 concentration in the circulating blood is increased. For example, MS patients typically indicate that they are not thirsty and drink simply because they know they have to drink.

#### 4.1.3 PVD and sleep behaviour

**Figure 7:**
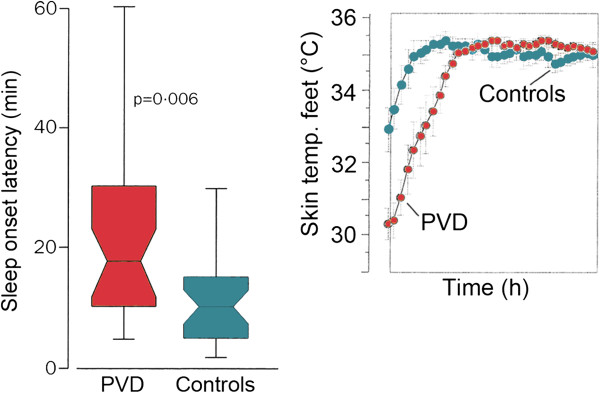
**Sleep onset in PVD patients.** (Left) Sleep onset time in PVD subjects is longer than in controls. This is due to the link between sleep onset and redistribution of temperature (modified from [90], with permission). (Right) Mean foot skin temperature before and during sleep. The delay in warming up of the feet explains the delay in sleep onset time (Courtesy of Kräuchi K).

PVD subjects often indicate that they require a long time to fall asleep, particularly when their feet are cold. Indeed, studies have shown that body temperature and sleep are interrelated [90]. Sleep is typically initiated when central body temperature is declining and body heat loss on the extremities is maximal. This indicates that we fall asleep only after our extremities, particularly our feet, are warmed up, and this takes longer on average in subjects with PVD (Figure 7). Melatonin is one factor involved in the fine-tuning of vascular tone in selective vascular beds [91], and PVD subjects have circadian phase delay of dim-light melatonin onset [92].

#### 4.1.4 PVD and physical activity

Physical activity and mechanical stimuli can, in some PVD subjects, provoke local vasospasm. Examples are algodystrophy or acute posttraumatic ischaemic of the limbs [93], exercise-induced amaurosis fugax-like symptoms [94] due to vasospasm of the retinal arteries, painful and ischaemic mammilla provoked by nursing [95,96] or orgasmic headache due to transient vasospasm [97-99]. All these symptoms fortunately occur infrequently. Most PVD subjects tolerate physical activities and are physically even more active than non-PVD subjects, particularly in endurance sports like cycling or running.

#### 4.1.5 PVD and professional activity

Subjects with PVD are generally very meticulous. They tend to be very exact and diligent, particularly at work. They are often successful in their careers. Interestingly, there are more PVD subjects amongst the academics than amongst blue-collar workers. We observed only very few farmers or gardeners with PVD syndrome. Furthermore, we made the observation that black Africans developed the symptoms of PVD only after moving from Africa to Europe. At present, we do not have an explanation for this relationship. One hypothetical explanation is light exposure. Vitamin D may play a role, but has not yet been studied.

#### 4.1.6 PVD and sensitivity

**Figure 8:**
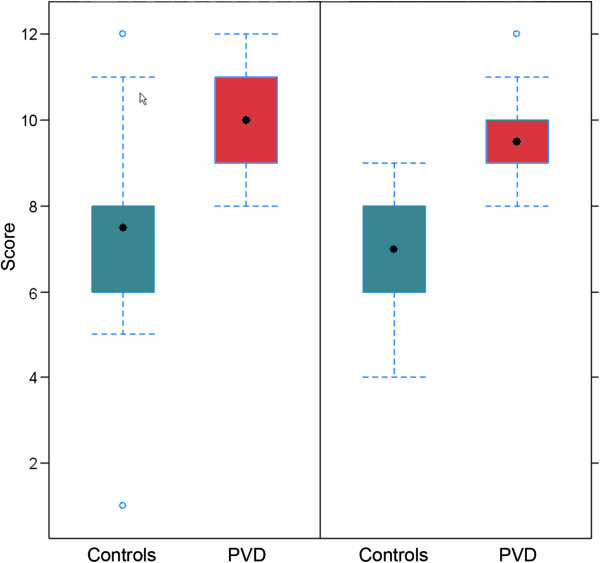
**PVD subjects are good smellers.** ‘Sniffin' Sticks’ score in healthy (left) and normal-tension glaucoma patients (right) with and without PVD. (Modified from [100], with permission).

**Figure 9:**
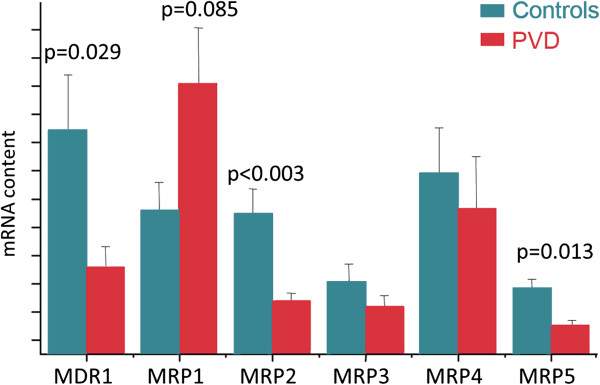
**The role of transport proteins: MDRI (P-glycoprotein) and MRP (multidrug resistance-associated protein).** The mean expression of these genes is significantly different in PVD subjects in relation to healthy controls. (Modified from [101], with permission).

Based on clinical experience, PVD subjects are generally more sensitive. PVD subjects, for example, are able to smell odours that others cannot [100] (Figure 8), and they do have a higher pain sensation. Some PVD subjects perceive thunderstorms as well as *foehn* weather situations stronger than non-PVD subjects. It is our clinical experience that PVD subjects also have a higher sensitivity to certain drugs. They tolerate these drugs well but often only when given at much lower doses. This might partly be explained by the different expressions of certain ATP binding cassette (ABC) transporter proteins [101] (Figure 9).

The vascular response to nicotinates is increased [102]. We observed a young PVD patient who lost the tips of his fingers and nose after receiving an infusion containing adrenalin to treat arterial hypotension, which occurred in the context of an infection. Although the clinical observations are quite consistent, all these aspects have not yet been studied sufficiently.

#### 4.1.7 PVD and psychological stress

Not much is known about the stress level of people with PVD. However, they tend to react differently to stress, particularly by reducing BF to the extremities and to the eyes [103]. In extreme situations, this can even lead to an infarction (e.g. an infarction of the ONH). We observed ONH infarctions in two stock market traders when share values dropped dramatically and in one student who failed an exam. A schoolgirl suffered from a retinal arterial branch occlusion after a major stress. We often observed retinal venous occlusion in PVD subjects after major psychological stress (see ‘Retinal vein occlusion’). In some subjects with PVD, an increased heterogeneity of skin perfusion, e.g. in the face, can be observed by eye but even better with the help of a thermal camera. Emotions can also provoke reversible cerebral vasoconstrictions [104].

#### 4.1.8 PVD and pain

We already discussed pain sensitivity. Some PVD subjects also suffer from muscle cramps and other pain. Sometimes they feel pain deep in their eyes (behind the upper eyelid), which most likely is due to ischaemic of the ciliary muscle. These eye pains vanish quickly after treatment with a calcium channel blocker (CCB) such as nifedipine.

#### 4.1.9 PVD and migraine

PVD and migraine are not identical and should not be confounded. Although PVD subjects suffer more often from migraine and vice versa, the two entities are principally independent [105]. While patients with migraine often have thermal hypersensitivity between attacks [106], a migraine attack can trigger the symptoms in a PVD subject, such as cold hands in the phase of aura. A reduction of neurovascular coupling during the acute phase has also been reported, potentially explaining why migraine patients avoid both intensive light and noises [107,108].

**Figure 10:**
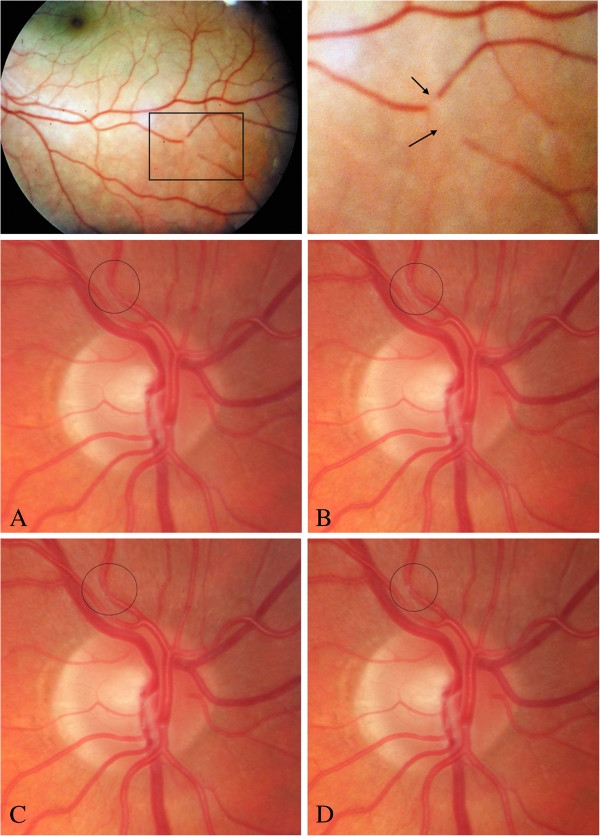
**Retinal migraine.** Fundus photograph taken during an attack of a retinal migraine (top). Area of focal dynamic spasm along the right superior temporal branch retinal artery. Photographs (**A**, **B**, **C**, **D**) represent different phases of the cycle (middle and bottom). (From [57] (top) and [112] (middle and bottom), with permission).

PVD subjects sometimes - but not often - suffer from retinal migraine, also called ‘presumed retinal vasospasm’ [109]. Retinal ‘angiospasm’ during migraine has already been described in 1910 [110]. In 1939, a relationship between retinal vasospasm and alterations in the nailfold capillaries was described [111]. Such focal retinal arteriolar vasospasm can be dynamic with spastic cycles of a few seconds [112] (Figure 10). The reversible vasoconstriction in the retina [113] indicates that there must exist stimuli for vasoconstriction other than the autonomic nervous system. Taking neurovascular coupling into consideration, it seems feasible that a spreading depression [114] acts as a trigger factor for the vasoconstriction.

#### 4.1.10 PVD and altitude sickness

Altitude sickness is a general term encompassing a spectrum of disorders that occur at higher altitudes. Most individuals suffer from altitude sickness to some extent when they reach high altitudes within a short time without giving their body enough time to adapt. Potential symptoms are as follows: headaches, lack of appetite, nausea, vomiting, fatigue, dizziness, sleep disturbances and peripheral oedema. The eyes are often involved. BF disturbances and retinal bleeding may occur. There is evidence for a genetic basis for altitude sickness [115].

The major cause of altitude sickness is the low oxygen level at higher elevations that leads to tissue hypoxia and thereby to an increase of HIF-1α. This induces increased expression of several hormones, including erythropoietin and also ET-1. Carbonic anhydrase inhibitors, endothelin antagonists and CCBs alleviate the symptoms. In the long term, our body acclimates to this situation, for example, by building more red blood cells. Based on our still-ongoing studies, we can state that the symptoms of altitude sickness are more pronounced in PVD subjects. We observed a young female subject with a known PVD syndrome who lost consciousness on a hot air balloon ride over the Alps. At high altitude, we also observed an increase in the retinal venous pressure, which is most likely a consequence of the rise of ET-1 [116].

#### 4.1.11 PVD, tinnitus and sudden hearing loss

PVD subjects with NTG often indicate suffering from tinnitus and remarkably often indicate a history of sudden (mostly reversible) hearing loss [117]. Hearing problems in the context of migraine have been described [118], but the relationship between hearing and PVD needs to be studied. Nevertheless, the presence of ET receptors in the spiral modiolar artery makes a relationship probable [119]. The majority of patients with sudden hearing loss, despite apparent good health, have signs of disturbed microcirculation in the eye [120]. In addition, auditory processing deficits occur more often in individuals with primary open-angle glaucoma [121], supporting the concept of glaucoma as a sick eye in a sick body [122].

#### 4.1.12 PVD and thyroid dysfunction

Hyperthyroidism may induce coronary vasoconstriction [123,124], and treatment with l-thyroxine can induce coronary spasm [125]. Hypothyroidism also can be associated with vascular dysfunction such as impaired endothelial- and non-endothelial-mediated vasodilation [126]. Hypothyroidism is associated with NTG [127], and interestingly, we find thyroid antibodies in most of these NTG patients. The vast majority of such patients are PVD subjects. An association between Raynaud's phenomenon and hypothyroidism was also described [128]. The causal relationship is not yet clear. Theoretically, ischaemic lesions in the thyroid gland may stimulate an autoimmune process or, conversely, autoimmunity may cause secondary vascular dysfunction.

### 4.2 General signs of PVD

In addition to the above illustrated symptoms, several signs characterise PVD subjects. These signs, which can be quantified objectively, will be summarised in the following paragraphs.

#### 4.2.1 PVD and temperature

**Figure 11:**
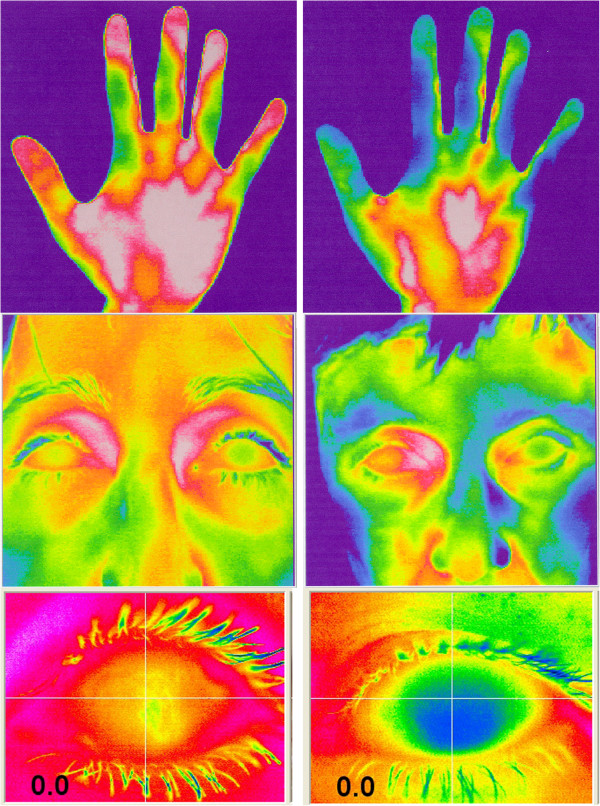
**Examples of thermography of the hands, faces and eyes of controls (left) and PVD subjects (right).** (From [57], with permission).

**Figure 12:**
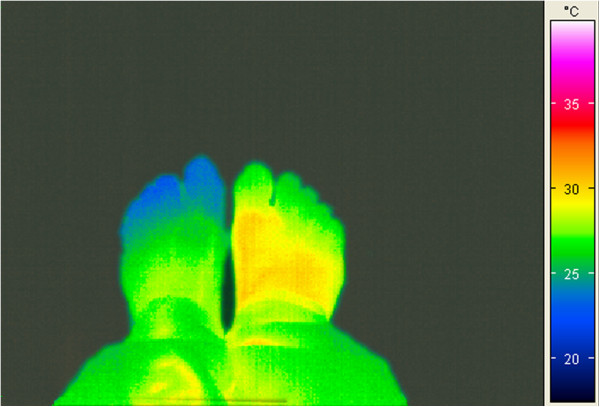
Thermography picture of the feet of a PVD subject with asymmetric temperatures.

The sensation of cold extremities, a leading PVD symptom, is confirmed by finger skin temperature measurements [29,87]. PVD subjects are characterised by a lower distal (hands and feet) skin and corneal temperature (Figure 11). In some PVD subjects, the temperatures are asymmetric (Figure 12).

However, proximal skin temperatures of PVD subjects do not differ significantly from other subjects. Furthermore, in the circadian rhythm, PVD subjects also show a phase delay in foot skin temperature in comparison to non-PVD subjects [129]. Reduction of temperature due to vasospasm can occur anywhere on the body surface, not only on the hands, feet or nose but also at the scrotum [130] or the mammillae [131]. Interestingly, corneal temperature correlates well with BF of the ophthalmic artery [132,133] and with finger temperature even after adjusting for environmental and tympanic temperatures and for age and sex [44].

#### 4.2.2 PVD and endothelin-1

**Figure 13:**
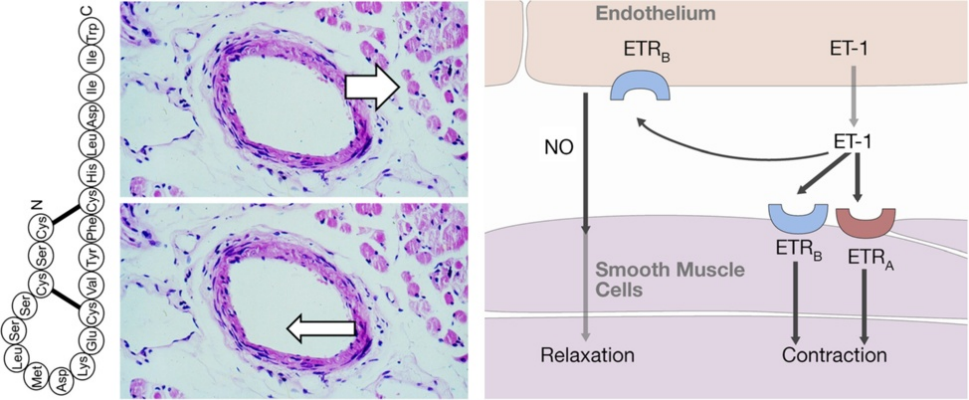
**Endothelin-1.** ET-1 is a 21-amino acid peptide (left) secreted from endothelial cells predominately abluminally and to a lesser extent intraluminally (middle). Endothelin receptor A (ETR_A_) is located on smooth muscle cells and mediates vasoconstriction whereas ETR_B_ is on both the smooth muscle cells and the endothelial cells and has a more neutral effect. (Modified from [83], with permission).

**Figure 14:**
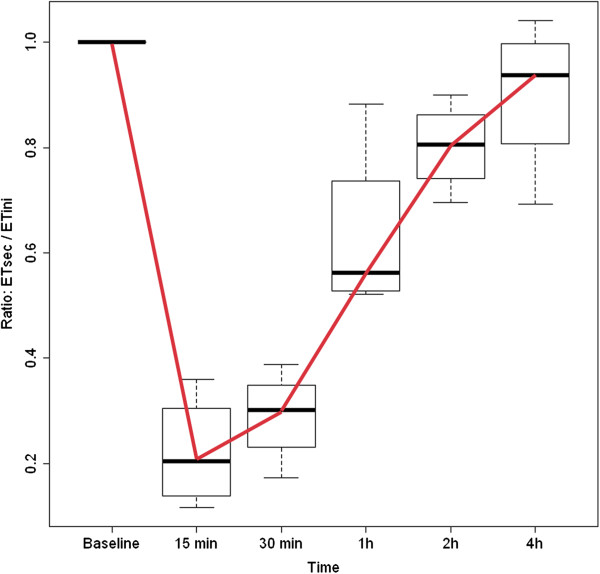
**Refractoriness to ET-1.** After the first stimulation by ET-1, subsequent stimulations lead to a smaller response. The first response is arbitrarily defined as 1, while subsequent responses are less than 1. The vessels recover within about 4 h. (From [134], with permission).

ET-1 is a peptide produced mainly, but not only, by endothelial cells. It is secreted abluminally to regulate local vascular tone. A small part is secreted intraluminally, leading to a certain concentration of ET-1 in the circulating blood (Figure 13). Production of ET-1 in other cells, particularly under pathological conditions (see ‘Secondary vascular dysregulation’), further contributes to the plasma level. After the binding of ET-1 to its receptor, the complex is internalised into the cell. A major stimulation by ET-1 is therefore followed by a refractory phase (Figure 14).

Circulating ET-1 is mainly eliminated in the lung. Plasma level of ET-1 therefore reflects the balance between production and elimination of ET-1 and thus represents, to some extent, local activities. In healthy PVD subjects, ET-1 is slightly increased [27]. Both increased [135-138] and normal circulating ET-1 plasma levels [139] have been described in NTG patients. In one study, ET-1 was higher in those with progressive ONH damage than in those in which the damage has stabilised [140]. Other authors found a normal ET-1 level at baseline, but an abnormal response with changing body position [141] or after cold provocation [142]. There are conditions in which local ET-1 concentrations do not correspond to the plasma level, for example, in the aqueous humour of glaucoma patients [139]. Notably, ET-1 in the aqueous humour correlates with IOP but not with systemic ET-1 plasma level [143]. Thus, in NTG patients, ET-1 may have a local as well as a systemic role, while in high-tension glaucoma (HTG) patients ET-1 may be dominantly increased to ocular tissue. Overall, for an adequate vascular function, the balance between vasoconstrictors (particularly ET-1) and vasodilators (particularly NO) [39] is of importance. In addition to the local concentration of ET-1, the local sensitivity to ET-1, which in turn depends on the number of ET receptors, is of importance as well [134,144]. Receptor density is locally increased in patients with giant cell arteritis. Interestingly, ET sensitivity is also dependent on blood pressure (BP). PVD subjects with low BP have higher ET-1 sensitivity [145]. Polymorphism of the ET receptor A seems to be associated with NTG [146]. A variant of the ET receptor A gene also modulates the risk for migraine [147]. Corresponding studies with healthy PVD subjects have not been done yet.

#### 4.2.3 PVD and blood pressure

**Figure 15:**
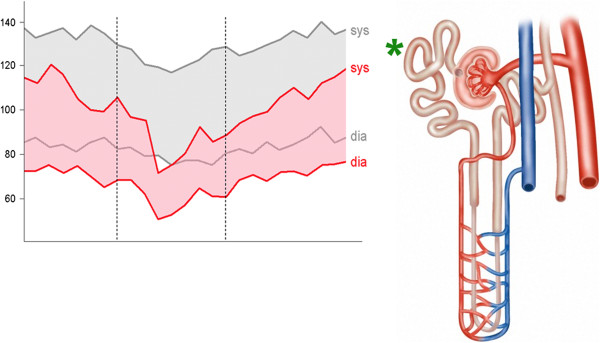
**Daytime BP of glaucoma patient with PVD.** (Left) Example of a 24-h blood pressure profile of a PVD subject (red) in relation to a control subject (grey). (Right) The reabsorption of sodium in the proximal tubule of the kidney (asterisk) is most probably reduced by the activation of PG E2 via ET-1. (Modified from [57], with permission).

Aside from cold extremities, low BP is a characteristic of PVD subjects, especially when they are young. The diurnal pattern of the mean arterial pressure nearly mirrors the distal minus the proximal skin temperature gradient, providing a measure for distal skin BF. In other words, diurnal BP variations are associated with changes in distal-proximal skin temperature gradients. Therefore, subjects with cold extremities (particularly PVD subjects) mostly have lower BP than control subjects [148]. Glaucoma patients with PVD also have lower mean daytime BP (Figure 15).

**Figure 16:**
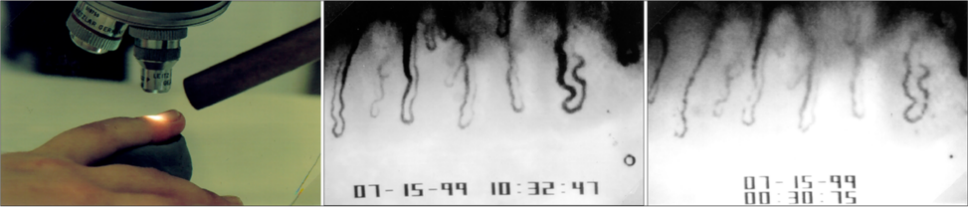
**Nailfold capillary microscopy with a cooling device.** The nailfold is made transparent by a drop of oil and is then videotaped (left). Normal blood flow (middle) and flow stopping after cold provocation (right). (Modified from [57], with permission).

 BF standstill in the nailfold capillaries is often used as the gold standard for the diagnosis of PVD (Figure 16). Interestingly, BF standstill in the nailfold capillaries after cold provocation was negatively correlated with the lowest systolic BP at night (dipping) [149]. Conversely, patients with nocturnal over-dipping had reduced retrobulbar BF with increased resistivity [150]. Systemic hypotension and PVD occur more often in young females than in postmenopausal women or in men. It was suggested that local β-adrenergic vasodilatation may offset α-adrenergic vasoconstriction in women to a greater extent than it does in men [151]. NTG patients have reduced sodium reabsorption in the proximal tubule, in spite of a low BP [152], probably also influenced by the slightly increased ET-1 level [153].

#### 4.2.4 PVD and the heart

The role of vascular dysfunction in the development, progression and prognosis of atherosclerosis has been well documented [8]; however, vascular dysfunction is not pathognomonic for atherosclerosis. Chest pain or ischemia despite structurally normal, non-atherosclerotic coronary arteries is quite common, particularly in women, and may represent local dysregulations of the epicardial or the microcirculatory coronary vessels. Coronary vasospasms of apparently healthy arteries were described by Prinzmetal in 1959 [154]. Such spasms of the epicardial arteries occur at rest or after certain provocations, sometimes triggered by psychological stress [155]. Interestingly, the typical reduction or even stop in BF in the nailfold capillaries of PVD subjects in response to local cooling is also seen in patients with vasospastic angina [54]. Furthermore, vasospastic angina is far more common in Asian countries [156], a pattern similar to both PVD and NTG [157]. Microvascular angina, particularly present in women, is another entity associated with dysfunctional vessels, particularly in the microcirculation [158]. However, it more likely affects elderly women with cardiovascular risk factors, thus a population different from those with PVD. However, microvascular angina may be an umbrella term for several vascular diseases, and PVD may be responsible for symptoms in a subset of these patients, especially in those where an abnormal increased pain sensitivity is proposed to be the pathomechanism [159]. An interesting observation in PVD patients is that electrocardiogram changes typical for myocardial ischemia occur frequently when patients are monitored over 24 h, thus silent ischemia could take place in these patients, particularly at night [160,161]. Interestingly, disorders associated with secondary elevation of ET-1 and SVD, such as rheumatoid arthritis or psoriasis, show endothelial dysfunction and a significantly higher cardiovascular mortality [162,163].

#### 4.2.5 PVD and the autonomic nervous system

**Figure 17:**
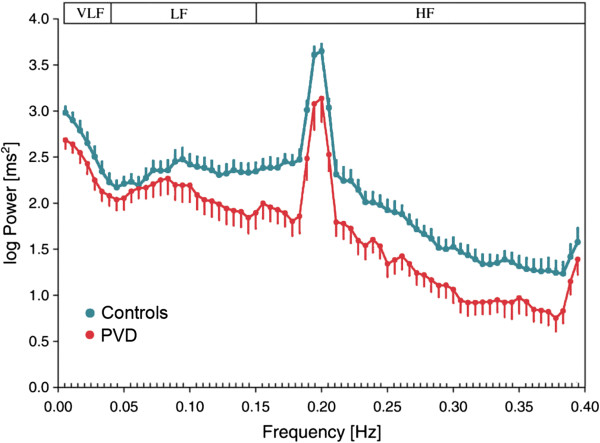
**Power spectrum of heart rate variability.** (Red) Average of PVD subjects and (blue) average of controls. VLF, very low frequency band; LF, low frequency band; HF, high frequency band. Paced breathing at 0.2 Hz. (Modified from [164], with permission).

Vascular dysregulation is often explained by autonomic nervous dysfunctions. Indeed, analysis of heart rate variability revealed an autonomic imbalance in healthy PVD subjects with sympathetic predominance [164] (Figure 17). This is in agreement with the observation of a shift in the sympathovagal balance of the autonomic nervous system of NTG patients towards sympathetic activity [165].

Furthermore, PVD subjects show an increased reactivity in the well-innervated choroid [166]. PVD subjects also show a temperature dysregulation in the extremities and in the sympathetically innervated skin. This also explains the influence of emotions (including anxiety) on skin perfusion [37,167]. This was indirectly confirmed by a study comparing slow and normal hand re-warmers. Slow re-warmers demonstrated less activity in the parasympathetic nervous system during the cold provocation protocol compared to normal re-warmers [168]. Dysfunction of the autonomic nervous system in NTG patients [169] was related to ET-1 levels [170].

The exact causal relationship between autonomic nervous dysfunction and vascular dysfunction is unclear. A dysfunction of the autonomic nervous system can induce dysregulation of vessels. On the other hand, vascular dysregulation leads to chronic intermittent hypoxia, and this in turn increases sympathetic nervous activity chronically [171]. The involvement of the non-innervated retinal vessel, however, demonstrates that although the autonomic nervous system is involved, it cannot be the only player in the pathophysiology of PVD.

#### 4.2.6 PVD and circadian rhythm

Circadian rhythm and thermoregulation are interconnected phenomena [172]. The human body consists of two compartments, the heat-producing core and the heat-losing regulating shell. Even in a comfortable thermo-neutral environment, heat produced by the core is transferred to the peripheral parts of the body, which serve the function of heat loss. These parts are distal skin regions - fingers and toes. Heat loss is autonomically regulated and occurs via constriction or dilatation of peripheral blood vessels [173].

**Figure 18:**
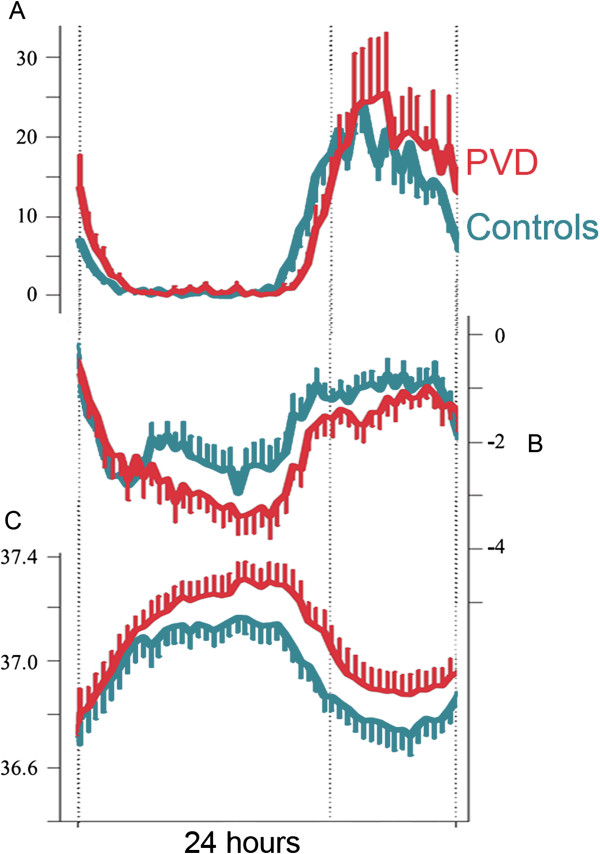
**Phase delay of the circadian system in PVD subjects in relation to normal controls.** (**A**) Melatonin concentration in the saliva, (**B**) temperature gradient (distal minus proximal skin temperature) and (**C**) core body temperature within a 24-h period without sleep. The delay is approximately 1 h. (Modified from [92], with permission).

There is a close link between body heat loss and sleep induction. When falling asleep, the core body temperature declines, while distal skin temperature increases, indicating heat redistribution from the core to the shell. The degree of peripheral blood vessel dilatation of the skin of the hands and feet is a good physiological predictor for the rapid onset of sleep [174]. There is a strong association between thermal discomfort from cold extremities and sleep onset latency in the general population [29]. It has been shown that PVD subjects with sleep onset insomnia exhibit a phase delay of the circadian system (circadian rhythms of distal and proximal skin temperatures, core body temperature and salivary melatonin secretion) by approximately 1 h, but no differences in overall sleep times [92] (Figure 18).

#### 4.2.7 PVD and gene expression in lymphocytes

**Figure 19:**
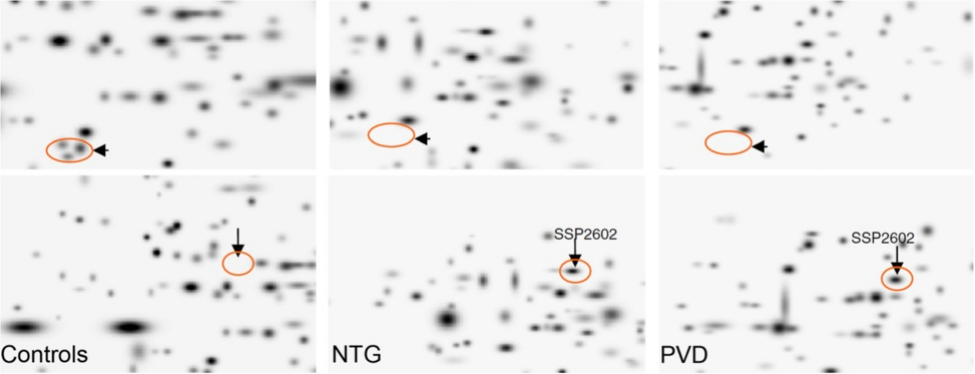
**Differential proteomics imaging of circulating lymphocytes.** (Top) A pathology-specific protein cluster is completely suppressed both in NTG patients and in healthy PVD subjects versus controls. (Bottom) The marked protein (SSP2602) is highly upregulated both in NTG patients and in healthy PVD subjects compared to the expression profile of controls. (Modified from [176], with permission).

Cells adapt their gene expression depending on internal and external information. Gene expression therefore reflects, at least to some extent, the environment in which these cells live. Circulating lymphocytes are not only easily accessible but also circulate through the whole body and are therefore good candidates to compare populations. Indeed, the gene expression profiling of PVD subjects revealed significant differences from non-PVD subjects but is very similar to that of glaucoma patients [175,176] (Figure 19). The genes and gene products of interest as potential diagnostic and drug targets have been summarised in other review articles [177,178].

#### 4.2.8 PVD and oxidative stress

**Figure 20:**
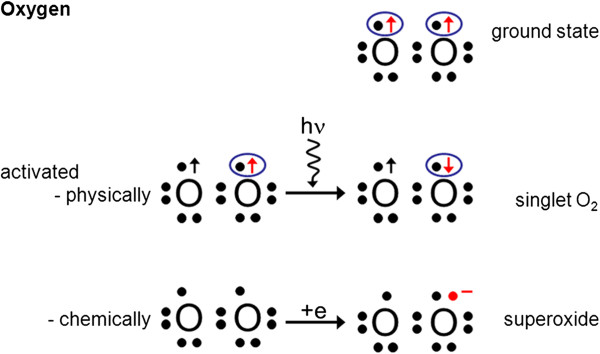
**Activation of ground state oxygen.** Ground state oxygen is relatively inert due to a parallel spin of two free electrons (spin restriction). Oxygen can be activated by adding energy, turning one spin. This leads to singlet oxygen. Oxygen can also be activated by adding one single electron, leading to superoxide. This is particularly the case in the mitochondria during reperfusion.

Reactive oxygen species (ROS) play a major role in both physiology and pathophysiology. If production of ROS exceeds its elimination capacity, oxidative stress results. Oxidative stress can be local or more generalised. Because PVD leads to unstable BF and thus to an unstable oxygen supply in certain organs, ROS productions may be triggered (Figure 20).

**Figure 21:**
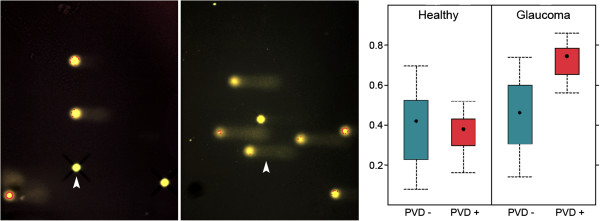
**One possibility to estimate oxidative stress is DNA break quantification by comet assay analysis.** (Left) Example of lymphocytes of a healthy subject. (Middle) Lymphocytes of a patient with glaucoma and PVD. (Right) Mean number of the breaks (quantified as tail moment) demonstrating an increase of DNA breaks in subjects suffering from both glaucoma and PVD. (From [180], with permission).

The majority of PVD subjects, especially when they are young, can cope with oxidative stress and do not develop tissue damage. Patients suffering from both PVD and glaucoma show a higher number of DNA breaks in their circulating lymphocytes (quantified by comet assay), indicating that oxidative stress is present even systemically [179,180] (Figure 21). In glaucoma, oxidative stress occurs mostly in the mitochondria of the retinal ganglion cells and their axons. Research, therefore, is focused on developing strategies to maintain mitochondrial function [181].

## 5 Visual signs and symptoms of PVD

### 5.1 PVD and visual function

**Figure 22:**
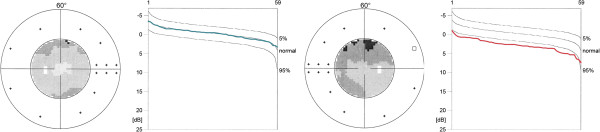
**Examples of visual fields (Octopus program G1) presented by greyscales and the corresponding Bebie curves.** (Left) Healthy control and (right) healthy PVD subject.

Most healthy subjects with PVD do not complain of visual disturbances, though some rarely indicate scintillation. In contrast to this perception, quantitative perimetry shows fluctuating visual defects in many subjects with PVD [30,45,182,183]. These are commonly diffuse defects best visualised by the Bebie curve [184] (Figure 22).

However, some researchers have first questioned whether changes in OBF lead to changes in visual function. Experimental studies in a pressure chamber then revealed that mean visual sensitivity is indeed directly and quickly influenced by oxygen saturation [185]. Although not felt, the differential light sensitivity decreased as oxygen concentration dropped. Visual fields reveal short- [186] and long-term [187] fluctuations. Certainly, long-term fluctuation is increased in PVD subjects [188]. Interestingly, if glaucoma patients with PVD show visual field defects, then only the diffuse component of the defect fluctuates [1,189].

### 5.2 PVD and ocular blood flow

It is known that the circulation in the extremities or the heart may react with vasospasm [156]. Rather surprising is the fact that the eye can react with inadequate vasoconstrictions as well. In 1910, Blessing and Amburger described retinal angiospasms during migraine [110]. In 1939, Lisch described functional vascular dysregulations of the eye and recognised a relationship with a dysregulation of the finger capillaries [111]. In 1948, Traquair observed spasm in the central retinal artery [190]. Incidentally, at that time, the visual symptoms of Adolf Hitler were interpreted by his physician as being of vasospastic origin [191]. In 1986, we described an involvement of the eye in primary vasospastic syndrome (we call it PVD today) [182,183]. We also described the assumption that vasospasm might be involved in the pathogenesis of glaucomatous damage [192] and discussed the potential role of CCBs to treat this condition [193].

At that time, it was difficult to measure OBF in humans, and our suppositions therefore were mainly based on the quantitative relationship between visual function and peripheral BF. The measurement of OBF is still challenging [194], and reliable methods have been made available only recently [4,195]. From a historical prospective, it is interesting that the hypothesis of PVD affecting OBF was based on the analogy of nailfold capillaries [2] and visual field behaviour [188,196]. In PVD subjects (but not in others), there was a significant correlation between nailfold capillary BF and visual fields. After cold provocation, both the nailfold capillary BF and the visual field deteriorated; likewise, after an intake of CCBs [197], both nailfold capillary BF and the visual fields improved.

It was debated as to what extent the observed visual field improvement in PVD subjects after treatment with a CCB may be due to the improved OBF or rather a direct effect of these drugs on neural tissue [198]. However, a similar visual field improvement has been observed with other OBF-improving drugs, such as with carbonic anhydrase inhibitors [199] and even with CO_2_ breathing [200]. It has also been shown that the same people responding well to CO_2_ also respond well to CCBs; additionally, subjects responding well to CCBs in the short term also respond well in the long term [201]. In contrast to non-PVD subjects, in PVD subjects, OBF is directly related to ocular perfusion pressure [202,203], indicating a disturbed autoregulation [204] of ocular perfusion in PVD subjects. Although BF velocity in the periphery [2] and in the ocular circulation is decreased on average, BF in individual PVD subjects can be normal under baseline conditions. However, a cutaneous cold provocation induces an immediate decrease in retinal and ONH perfusion, and this decrease diminishes or disappears quickly when the hand is immersed in warm water [205]. In PVD subjects examined with colour Doppler imaging, the peak systolic and end diastolic velocities and the resistivity index of the central retinal artery significantly correlated with the mean ocular perfusion pressure [206]. In glaucoma patients with PVD, BF in the choroid and ONH is reduced compared to glaucoma patients without PVD [207]. More details will be discussed in the section 6.1.

### 5.3 PVD and retinal vascular spatial irregularities

Healthy PVD subjects showed higher spatial irregularities in retinal vessels than non-PVD subjects [208]; in other words, retinal vessels of vasospastic subjects are less smooth. This indicates that the mechanism of dysregulation (particularly endothelium dysfunction) is not homogenous along the vessels. It rather affects different parts of the same vessel stronger than other parts. This irregularity is not identical to overall retinal arteriolar narrowing, which, interestingly, is an important risk factor for open-angle glaucoma [209].

### 5.4 PVD and stiffness of retinal vessels

Vessels become stiffer when they become sclerotic. Interestingly, pulse wave propagation in the retinal vessels of PVD subjects is faster, indicating that they are stiffer without recognisable morphological alterations [210]. This is also the case in untreated NTG patients [211].

### 5.5 PVD and neurovascular coupling

**Figure 23:**
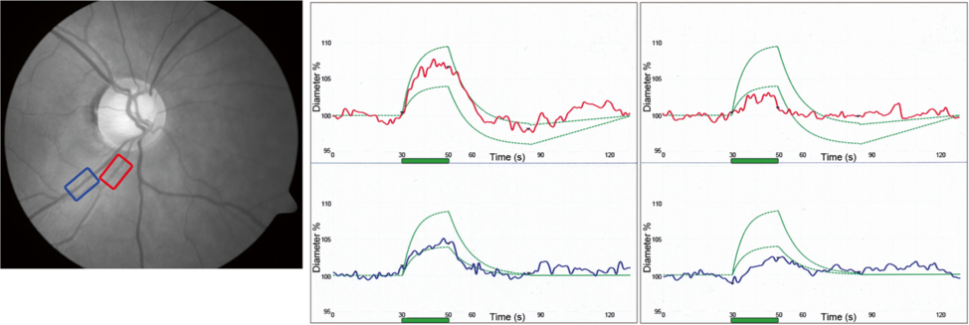
**Retinal vessel analyser registers the size of a selected retinal artery and vein.** Flickering light (green bar) leads to vasodilation of arteries (red) and veins (blue) in healthy subjects (middle) and to a lesser extent in subjects with vascular dysregulation (right). The green curves indicate the normal range. (Modified from [83], with permission).

Flickering light to the retina leads to a dilation of both retinal arteries and veins within seconds (Figure 23). This response is reduced in healthy PVD subjects [11]. The cause of this is not yet clear. Most probably, it is mainly due to an altered function of the vascular endothelial cells in PVD subjects (see ‘The role of vascular endothelial cells’). Interestingly, the general vessel response to flickering light was also decreased in POAG patients [12].

### 5.6 PVD and the blood–brain barrier

The central nervous system (including the retina) is a very sensitive system. Specialised structures, such as tight junctions between endothelial cells, are required to maintain a stable environment and to ensure appropriate neuronal activities [212]. The barrier in the ONH, however, is physiologically incomplete as a result of (a) diffusion from the fenestrated capillaries of the choroid into the ONH (see Figure 3) and (b) a nonspecific permeability of the capillaries, possibly mediated by vesicular transport [24]. The blood-retinal barrier is highly regulated, and this involves molecules such as ET-1 that are also involved in the regulation of vascular tone [213]. Expression of ET-1 is increased under hypoxic conditions, either locally in the retina, affecting retinal vessels, or systemically, thereby influencing the barrier in the ONH and adjacent retina by diffusion from the choroid. PVD can lead to hypoxia and thereby indirectly to barrier dysfunction. This is of special interest for glaucoma [26,178].

### 5.7 PVD and activation of astrocytes

**Figure 24:**
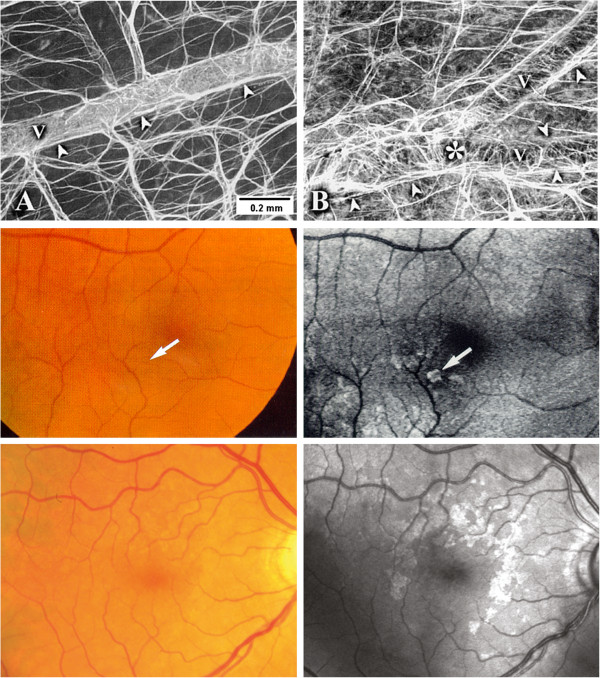
**Glial cell activation.** (Top) Activation of glial cells in the retina. Astrocytes connecting vessels with neurons (left) and irregular pattern of activated astrocytes (right). (Middle and bottom) Activated astrocytes increase light scattering, visible in red-free light (right), but not visible in colour photos (left). (From [214] (top), [215] (middle) and [216] (bottom), with permission).

Glial cell activation is a typical response to injuries of the central nervous system including the retina. The activation of glial cells, well demonstrated by increased glial fibrillary acidic protein staining, has been described in the retina in response to light damage or mechanical stress and also to hypoxia. Retinal astrocytes and Mueller cells also become activated in glaucoma. This includes a change of both the function and the morphology of these cells (Figure 24).

We have first described the clinical correlate of astrocytes activation in the retina [215]. Activated astrocytes change not only gene expression but also their morphology, leading to an increased backscatter [83]. Therefore, glinting spots can be recognised in red-free photos or by time domain optical coherence tomography [217]. Interestingly, activated astrocytes can be particularly observed in glaucoma patients with PVD [216].

### 5.8 PVD and autoregulation

Autoregulation describes the capacity to keep BF in an organ within a certain range independently of perfusion pressure. This is also the case for the eye, particularly for the retina, slightly less for the ONH and only limited for the choroid. A disturbed autoregulation has been described both for healthy PVD subjects [206] and for glaucoma patients with progressive disease [202]. The main impact of such a deficient autoregulation is unstable oxygen supply with the consequences of oxidative stress [204]. The dysfunctional autoregulation in PVD subjects is the major link between PVD and glaucoma [28].

### 5.9 PVD and the relation between peripheral and ocular circulation

ONH BF was correlated to both finger BF and finger temperature in PVD subjects but not in others [218]. This can be explained by deficient autoregulation allowing systemic factors such as perfusion pressure to dominate. The situation is different in the choroid, which is less autoregulated and highly innervated. Here, the BF was higher in patients with PVD, probably to keep the temperature at the back of the eye constant despite a reduced BF in the peripheral environment [219]. Corneal temperature in an unselected population correlates with finger temperature [44]. Interestingly, OBF and finger BF changes induced by cold provocation were correlated in glaucoma patients but not in others [220]. Additionally, retrobulbar BF in glaucoma patients is also correlated with ONH BF [221].

### 5.10 PVD and retinal venous pressure

Retinal venous pressure (RVP) was once assumed to be more or less equal to IOP [222], except in patients with increased intracranial pressure [223]. After ophthalmo-dynamometry was introduced to measure RVP, this assumption could no longer be made [224]. RVP is increased [15], and spontaneous venous pulsations occur less often in glaucoma, especially in NTG patients than in healthy controls [225]. RVP is also increased in other conditions such as diabetic retinopathy or in the fellow eye of patients with retinal vein occlusion. In PVD subjects, RVP can be normal (equal to IOP); most often, however, it is slightly or sometimes even markedly increased.

### 5.11 PVD and optic disc haemorrhages

**Figure 25:**
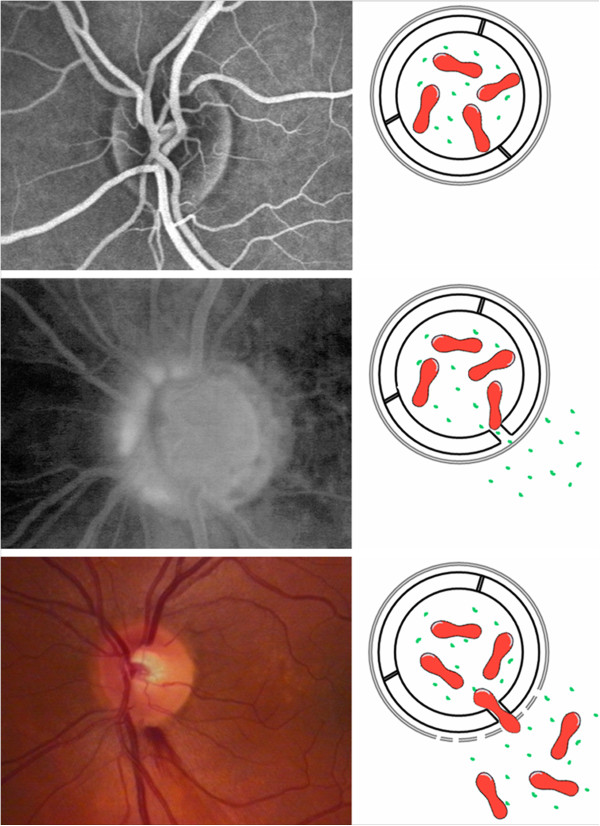
**Pathogenesis of optic disc splinter haemorrhages.** Under normal conditions, the vessels in and around the optic nerve head are watertight (top). If the barrier is opened at the level of the endothelial cells (e.g. by ET-1), small molecules such as water and fluorescein can leak out (middle). If at the same time the basal membrane in the same area is also weakened (e.g. by MMP-9), erythrocytes can also escape (bottom). (Modified from [26], with permission).

Optic disc haemorrhages (ODH) in the context of glaucoma were first described more than 100 years ago by Bjerrum in 1889, but studied in detail much later [226,227]. ODH are associated with morphological and functional progression of disease [228-230]. Interestingly, the presence of ODH is strongly associated with nail bed haemorrhages [231], indicating systemic factors involved in the pathogenesis of ODH. The fact that ODH occur more often in NTG [232] than in HTG patients and that they often precede damage and occur more often in females than in males provoked the question of a potential relationship between ODH and PVD. Indeed, ODH also occur in PVD subjects without glaucoma [233]. Furthermore, ODH occur in the contra-lateral eye of patients with retinal vein occlusion. We hypothesise that ODH are not a consequence of a disrupted vessel but rather of a dysfunctional blood-retinal barrier [26] (Figure 25). ET-1 seems to be involved not only in the pathogenesis of ODH but also in the pathogenesis of the increase of retinal venous pressure and retinal vein occlusion [84].

## 6 PVD and ophthalmic diseases

### 6.1 PVD and glaucoma

The pathogenesis of glaucomatous optic neuropathy and particularly the role of OBF has been discussed controversially [234] since the first description of optic nerve excavation by von Graefe in the nineteenth century. In addition to an increased IOP, the main risk factors for glaucomatous damage are IOP fluctuations [235,236], low BP [237,238], reduced perfusion pressure [239] and fluctuation of perfusion pressure [240]. While it was difficult to measure OBF in the past, the present methods clearly reveal that OBF is reduced in glaucoma patients, particularly if the damage is progressing despite a normal IOP [241,242]. The reduction of OBF was often interpreted as being only secondary to the glaucomatous damage. This assumption was also supported by the fact that there is only a weak relationship between glaucomatous optic neuropathy (GON) and atherosclerosis. Today, we know that a reduction in OBF predicts future GON progression [243-245]. Furthermore, vascular dysfunction in glaucoma is not limited to the eyes [246]. These facts argue in favour of a primary component of BF reduction in glaucoma. In addition, it has become evident that OBF regulation is more important than the OBF at baseline condition and that fluctuations in OBF are particularly damaging [4,234]. Indeed, glaucoma patients react with an altered OBF response when challenged by changes in perfusion pressure [202], hypercapnia [247,248], hyperoxia, flicker light stimulation [12] or cold provocation [220,249]. These patients also have altered vasoreactivity to ET-1 [250] and an imbalance between the vasoconstrictor ET-1 and vasodilator NO in both plasma and aqueous humour [39,251]. The prevalence of silent myocardial ischaemic was also higher [160,161].

**Figure 26:**
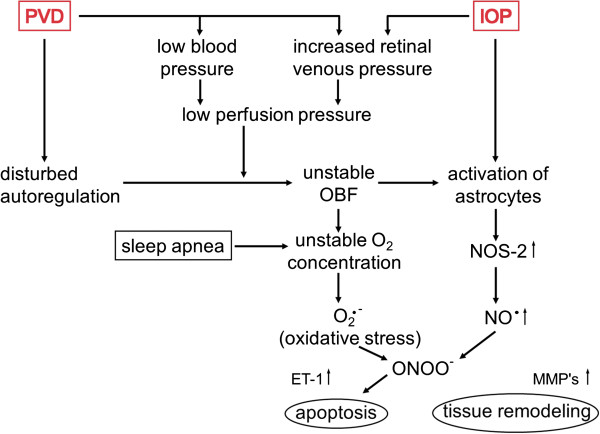
**There are two major risk factors for GON: PVD and IOP.** PVD decreases perfusion pressure by lowering blood pressure and by increasing retinal venous pressure. Increased IOP also reduces perfusion pressure and activates the astrocytes. PVD also leads to disturbed autoregulation and therefore to an unstable OBF. This in turn leads to oxidative stress. Oxidative stress together with activated astrocytes leads to the damaging peroxynitrite and cell death.

But what exactly is the cause of vascular dysfunction in glaucoma, and how does this contribute to damage? We postulated that PVD is the major cause of vascular dysfunction and formulated a concept of the pathogenesis of GON (Figure 26).

**Figure 27:**
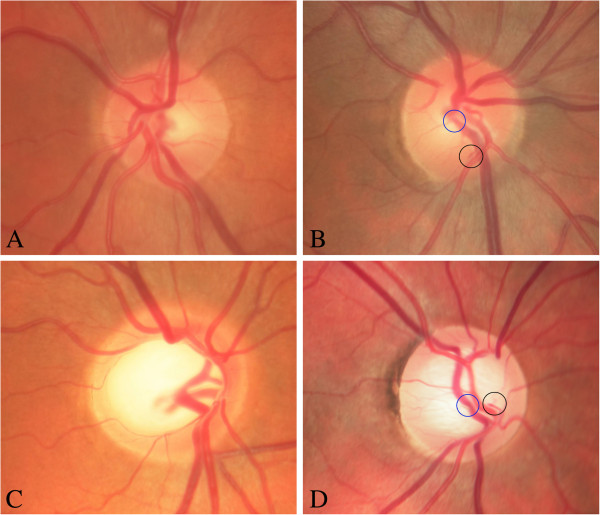
**Typical examples of optic nerve heads.** Taken from (**A**) a healthy subject, (**B**) a healthy subject with PVD, (**C**) an HTG patient (vessels are pushed towards the nasal quarter of the excavation) and (**D**) an NTG patient with PVD (vessels are not pushed to the nasal side, and the remaining neuroretinal rim is slightly pale). The rings highlight the local manifestations of vascular dysregulations.

While hypoxia per se may lead to some optic nerve atrophy, it is the instability of the oxygen supply, leading to oxidative stress, that contributes to glaucomatous atrophy [252]. Oxygen supply fluctuates if either the oxygen saturation of the blood fluctuates (e.g. in patients with severe sleep apnoea) or the OBF is unstable. OBF is unstable if either IOP fluctuates above or BP fluctuates below the capacity of autoregulation. However, even normal IOP and normal BP fluctuation lead to OBF fluctuation if the autoregulation itself is disturbed. This explains why PVD is a major risk factor for GON [3]. PVD is associated with (a) low BP, (b) increased retinal venous pressure and (c) disturbed autoregulation. Although these three conditions are interrelated [150], each can also contribute to the damage independently [253]. Figure 27 shows examples of optic nerve heads of (a) a normal healthy subject, (b) a healthy PVD subject, (c) an HTG patient and (d) an NTG patient. We abstain from a detailed description of the role of OBF in glaucoma in this review. For readers with interest in this field, we refer to the following literature: We summarised the role of vasospasm [1], systemic diseases [122], OBF [4] and autoregulation [204] in the pathogenesis of GON and presented our pathogenetic concept [254]. In a recent review, we further summarised the relationship between OBF and cardiovascular diseases [163]. The vascular aspects are also included in our glaucoma book [57]. Finally, the relationship between BF and glaucoma was described in detail in a pocketbook [255].

### 6.2 PVD and other eye diseases

#### 6.2.1 Retinal arterial occlusion

Retinal arterial occlusions normally occur in elderly patients with risk factors for atherosclerosis. Rarely, such occlusions occur also in young subjects without any signs of atherosclerosis [256]. It is sometimes explained by factors such as hyperhomocysteinemia [257], although the causal relationship remains unclear. Occlusions rarely occur during a migraine attack [258]. Most of these young and otherwise healthy patients with arterial occlusion suffer from PVD [1] and have slightly increased plasma level of ET-1 [81]; often, the occlusion is reversible [259].

#### 6.2.2 Retinal vein occlusion

**Figure 28:**
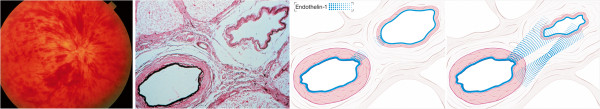
**Pathogenesis of retinal vein occlusion.** At the lamina cribrosa, the central retinal artery and central retinal vein are topographically very close and share a common adventitia (middle). This enables a molecular crosstalk between the two vessels (right). ET-1 (blue), for example, can diffuse from the ailing artery as well as from the adjacent hypoxic tissue to the very sensitive vein, leading to venous constriction. (Modified from [84], with kind permission from Springer Science + Business Media B.V.).

Retinal vein occlusion (RVO) occurs most often in elderly patients with vascular risk factors such as systemic hypertension and in patients with glaucoma. RVO, however, can sometimes occur in relatively young subjects without risk factors for atherosclerosis and without glaucoma. Most of these patients suffer from PVD [260]. We made the clinical observation that RVO in such PVD subjects most often occurred after a major stress episode. An association with both a cilioretinal artery occlusion [261] and with retinal arterial vasospasm has been described [262]. As in the older and more classical RVO patients [82], these young subjects also have increased ET-1 plasma levels [81]. We know from experimental studies that ET-1 injection in the vitreous body leads to transient complete obstruction of retinal vessels [85]. We hypothesised that RVO may primarily be caused by a local ET-1-induced constriction of the vein, either at the arterio-venous crossing in the retina or at the level of the lamina cribrosa. ET-1 diffuses either from the fenestrated capillaries of the choroid, from diseased arteries or from hypoxic retinal tissue to the vein [84] (Figure 28).

In contrast to what is often assumed, veins are not passive tubes but respond very sensitively to vasoactive hormones. Impaired generalised endothelial function in patients with RVO [263] has been reported, and this is compatible both with atherosclerosis of older patients and with PVD of younger subjects.

#### 6.2.3 Anterior ischaemic optic neuropathy

As with all arterial occlusions, atherosclerosis and its risk factors also play a major role for anterior ischaemic optic neuropathy (AION). Like retinal arterial occlusion and RVO, AION can occur in young subjects without any risk factors for atherosclerosis as well. We made the observations that the vast majority of these patients are PVD subjects and that the occlusions occur in most cases after major psychological stress [264,265] or, alternatively, are drug induced [266]. AION, however, can also be observed secondarily to other diseases (see ‘Secondary vascular dysregulation’) such as autoimmune diseases [267].

#### 6.2.4 Susac syndrome

**Figure 29:**
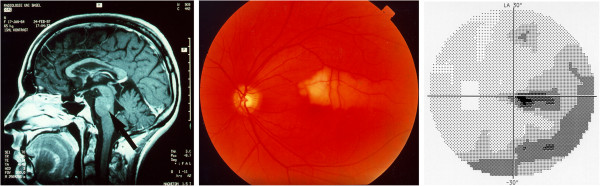
**Susac syndrome in a patient with severe PVD.** (Left) MRI of the pons with a hyperintensive lesion. (Middle) Localised retinal infarction. (Right) Corresponding visual field defects. (From [271], with permission).

The syndrome was named after J.O. Susac, who first described it in 1979 [268]. It is characterised by the clinical triad of encephalopathy, branch retinal artery occlusion [269] and sensorineural hearing loss. Like PVD, it mainly occurs in young women. It is generally assumed that an autoimmune process leads to damage and inflammation-related occlusion of the microvessels in the brain, retina and inner ear. Indeed, anti-endothelial cell antibodies could be detected in some patients [270]. It remains, however, unclear whether this is a primary or secondary event as, on the one hand, autoimmunity can lead to ischemia and, on the other hand, ischaemic lesions can induce autoimmunity. All patients we had the opportunity to examine suffered from the classical symptoms and signs of PVD [271] (Figure 29). As discussed with multiple sclerosis, further studies are necessary to sort out what came first in these Susac patients: the dysregulation or the autoimmunity (see ‘Causes of SVD’).

#### 6.2.5 PVD and optic nerve compartment syndrome

**Figure 30:**
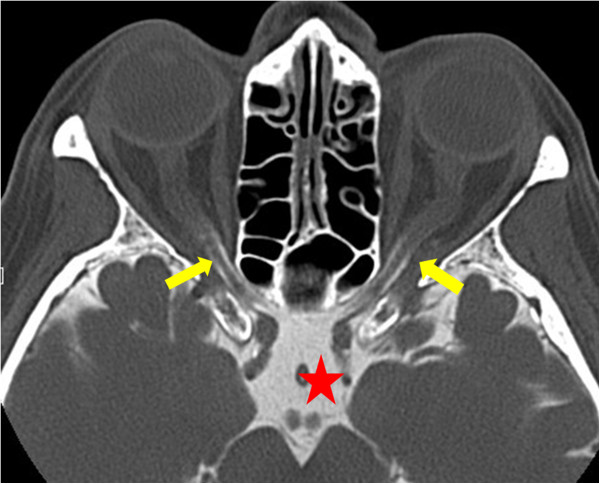
**Cisternography of a patient with optic nerve compartment syndrome.** Normal contrast-loaded cerebrospinal fluid in the pituitary cistern is indicated by a star. Stopping of the contrast-loaded cerebrospinal fluid in the posterior portion of the intraorbital subarachnoid space in both optic nerves is indicated by arrows (Courtesy of Killer HE).

Cerebrospinal fluid normally communicates freely throughout all CSF compartments. CSF sampled during lumbar puncture is therefore considered to be representative for all CSF compartments in terms of pressure and composition. However, in optic nerve compartment syndrome, there is proven segregation of CSF between the intracranial subarachnoid space and the subarachnoid space surrounding the optic nerve [272]. This compartment [273] is characterised by measurable differences of fluid composition [274], comparable to the composition of the subretinal fluid [275], reduced CSF exchange [273] (Figure 30) and extension of the optic nerve sheath diameter due to increased pressure [276,277]. These patients are often referred because of visual field defects. The visual field can be concentric constricted or more glaucoma-like, while visual acuity is often but not always reduced. The ONH is mostly more or less atrophic, sometimes glaucoma-like excavated [278] as we see in NTG [279] and rarely swollen [280]. The retinal venous pressure is nearly always increased.

The increase of CSF pressure leads to proliferation of meningothelial cells [281,282]. These cells participate in immunological processes in the CSF [283]. The change in the concentration of CSF proteins such as lipocalin-like prostaglandin-D- synthase could harm the neurons [284].

The pathogenesis of the compartment syndrome is not yet clear. A reduced outflow of the CSF through the lymphatic vessels may contribute [285-287]. It is our clinical experience that the majority of patients with optic nerve compartment syndrome are PVD patients. We have also observed that a treatment of PVD very often reduces the extension of the optic nerve sheath diameter and reduces retinal venous pressure in patients that had recently developed a compartment syndrome. In more advanced cases, steroids sometimes help, whereas in very advanced cases, only optic nerve sheath fenestration is helpful. We assume that the unstable oxygen supply due to PVD may lead to chronic inflammation via oxidative stress. This in turn could induce swelling and proliferation of the arachnoid trabeculae leading to a kind of valve allowing CSF to enter in the optic nerve compartment but hindering the backflow.

#### 6.2.6 Central serous chorioretinopathy

**Figure 31:**
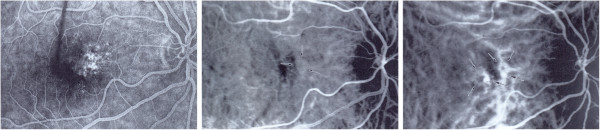
**Angiography of a 46-year-old female subject with recurrent central serous chorioretinopathy.** (Left) Disorder of the retinal pigment epithelium with leakage. (Middle) Slight congestion in the early capillary filling phase in indocyanine green angiography. (Right) Congestion of the draining veins clearly visible in the venous filling phase. (From [288], with permission).

Central serous chorioretinopathy is a common disorder characterised by accumulation of serous fluid under the neurosensory retina secondary to a localised defect of the outer blood-retinal barrier. The pathogenesis is controversial. We hypothesised that local vascular dysregulation in the choroidal BF plays a major role [288]. This assumption is based on the following observations: (a) indocyanine green angiography done during the acute phase revealed delayed filling and venous congestion in the corresponding area of the choroid most likely induced by venous constriction (Figure 31), (b) it is most often preceded by a major psychological stress and occurs more often in subjects with so-called type-A behaviour, and (c) it can be provoked by intravenous injection of epinephrine. The resulting hypoxia in the area of the corresponding lobulus may then secondarily break down the outer blood-retinal barrier.

We further observed that these patients were mostly PVD subjects and had increased ET-1 plasma level during the acute phase; in addition, they often showed increased retinal venous pressure. Interestingly, it occurs particularly often in young men. Male hormones most likely play a role. Once, a 45-year-old woman with a history of metamorphopsia was referred to our institution, and we diagnosed a classical central serous chorioretinopathy in a PVD subject under major professional stress. We told her that she was suffering from a disease we most often see in young men. On her second visit, she mentioned that she had been taking 40 mg of oral testosterone a day for the previous 2 months for better professional performance. Plasma testosterone level was markedly increased. We stopped testosterone treatment, and she recovered quickly and remained free of symptoms the following years [289].

#### 6.2.7 Leber hereditary optic neuropathy

Leber hereditary optic neuropathy (LHON) is a maternally inherited disorder resulting from point mutations in mitochondrial DNA (mtDNA) [290]. Ophthalmologic findings in LHON patients are variable, but classical LHON cases exhibit vascular tortuosity of the central retinal vessels, swelling of the retinal nerve fibre layer, a circumpapillary telangiectatic microangiopathy and a cecocentral scotoma with variable preservation of peripheral vision. The disease finally leads to optic disc atrophy. Despite a clear genetic background, other factors play a role in manifestation and progression for the following reasons: (a) not all patients harbouring a pathogenic LHON mtDNA mutation develop the disease, (b) patients live free of symptoms many years before the onset of the disease, and (c) only a few organs and particularly the visual system get sick. It is likely that internal or external factors additionally damaging mitochondria are contributing factors, such as smoking, excessive alcohol, light exposure [291] and age. We made the clinical observation that the majority of patients we saw with LOHN were PVD subjects. There are many possible interpretations of this: (a) it could be referral bias, (b) subjects with mitochondrial mutations have higher risk for PVD, or (c) PVD subjects have unstable OBF leading to oxidative damage of the mitochondria and thereby increasing the probability that mitochondria with pre-existing mutations decompensate. The relationship between LOHN and NTG is unclear, but the majority of NTG patients have no mtDNA mutations [292]. However, we saw a young man with PVD and acute unilateral LOHN in his right eye, who developed NTG in his unaffected left eye over the following 10 years.

#### 6.2.8 Retinitis pigmentosa

Retinitis pigmentosa (RP) encompasses a large group of hereditary diseases of the posterior segment of the eye characterised by degeneration, atrophy and, finally, loss of photoreceptors and retinal pigment epithelium, leading to progressive visual loss. Even though the disease has a genetic background, we assume that additional factors influence the manifestation of the disease. One potential modifying factor is disturbed OBF. Indeed, reduced OBF in RP patients has been described. BF is more or less always reduced in atrophic tissue, secondary to a decreased demand for supply. However, there are indications of an additional primary component mainly caused by PVD. We refer to a recent review [293].

## 7 Therapy

Although vascular dysregulation is frequent, often cumbersome and sometimes damaging, little research has been conducted to develop treatments. The present therapeutic armamentarium therefore is very limited and mainly based on clinical experience. The majority of PVD subjects do not need any treatment because they are healthy. Treatment should be considered if these subjects are bothered by signs and symptoms or if they show PVD-related disease like progressive NTG.

### 7.1 Lifestyle

To some extent, PVD subjects can reduce their symptoms by lifestyle modification. They should avoid, as far as this is possible, major emotional stress. In glaucoma patients with PVD, we often observed very fast progression of visual field defects during a major stress event, for example, in the context of a divorce. It has recently been shown that women who commonly suppress anger experience more thermal discomfort from cold extremities and have prolonged sleep onset latency [47]. Furthermore, PVD subjects should avoid extensive cold. We observed two PVD subjects that acquired sudden and irreversible bundle-shaped visual field defects during skiing. One woman with PVD repeatedly lost consciousness after jumping in cold water. PVD subjects are mostly slim, but we recommend that PVD subjects keep their BMI not too low and avoid major fasting periods. PVD subjects should not exaggerate with sport. We observed a fast progressive NTG in a young man bicycling every day for several hundred kilometres in a mountainous area. After he stopped doing this, his NTG stabilised. Daily mild physical activity, however, is helpful. PVD subjects should be careful climbing high mountains too quickly. Altitude sickness seems to be stronger in these subjects. We observed a young female ophthalmologist with marked PVD symptoms who lost her fingers and toes at high altitude. We further recommend avoiding strong light and wearing sunglasses. Similar to an unstable oxygen supply, light induces oxidative stress and damages the mitochondria [294-296]. We examined a gastroenterologist who had performed more than 22,000 endoscopies with his preferred left eye (this was before the advent of video monitors). In this left eye, he acquired colour vision deficiency, reduced contrast sensitivity and inhomogeneity of the pigment in the macular area, whereas the other eye remained normal [297].

### 7.2 Nutrition

We recommend a diet containing fruits and vegetables, particularly if they are rich in antioxidants such as anthocyanosides or flavonoids. Black currant anthocyanins have been shown to normalise abnormal levels of serum concentrations of ET-1 in patients with glaucoma [298]. Food rich in flavanols, particularly cocoa, may improve endothelial function in general [299]. Food rich in omega-3 fatty acids improves vascular regulation [300]. We also recommend green tea [301]. If BP is very low, salt intake should be increased [302]. For a more detailed description of our recommendation on nutrition, we refer to the book *Ocular Blood Flow and Glaucomatous Optic Neuropathy*[255].

### 7.3 Magnesium

**Figure 32:**
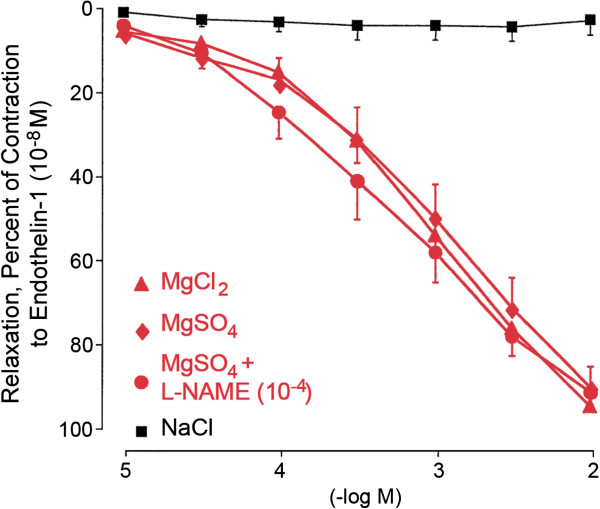
**Similar relaxing effect of increasing MgSO**_**4 **_**and MgCl**_**2 **_**concentrations added to ET-1 precontracted ciliary arteries.** (Modified from [303], with permission).

Magnesium is cheap and has few side effects. In *ex vivo* studies, it reduces the vasoconstrictive effect of ET-1 (Figure 32) [303] and potentially has a positive effect on visual fields [304].

### 7.4 Calcium channel blockers

CCBs are the classical anti-vasospastic drugs at present [197,305]. They reduce the effect of ET-1 [306,307] and improve visual fields of PVD patients [265]. It is not clear yet which of the CCBs is best and whether water-soluble or fat-soluble CCBs should be used. We use water-soluble CCBs like nifedipine for glaucoma and fat-soluble CCBs like nimodipine for retinal diseases. To reach the ONH of glaucoma patients, the drugs do not need to cross the blood–brain barrier. It is important to use very low doses [60] for the following reasons: (1) already low doses show efficient anti-vasospastic effects, (2) the drug sensitivity of PVD subjects is increased, and (3) in most cases, we do not want to further decrease BP. Nifedipine improves capillary BF in PVD subjects [308]. In a placebo-controlled 3-year study, nilvadipine slowed the visual field progression, maintained the optic disc rim and increased choroidal circulation in patients with NTG [309]. The better visual field preservation effect of betaxolol over timolol is most likely also due to the calcium antagonising side effect of betaxolol [310]. Topical flunarizine reduces IOP and protects the retina against ischaemic-excitotoxicity [311]. The use of CCBs in glaucoma has recently been reviewed [198,312].

### 7.5 Endothelin antagonists

Bosentan, a dual endothelin receptor antagonist, reduces coronary vasospasm [313], cerebral vasospasm [314] and pulmonary vasoconstriction [315] and increases OBF in patients with glaucoma [316]. Avosentan, an ET(A)-endothelin receptor antagonist, relaxes ciliary arteries [317]. Sulfisoxazole, another ET receptor antagonist, protects retinal neurons from the insult of ischaemic/reperfusion [318]. Nevertheless, a routine use of ET blockers at present is not or not yet recommended for treatment of PVD due to potential side effects.

### 7.6 Fludrocortisone

The rare PVD patients who have extremely low BP, not responding to salt intake, we treat with the mineralocorticoid fludrocortisone [319] at very low doses (0.1 mg twice a week). Even when only a slight BP increase is achieved, haemodynamic parameters are improved [320].

### 7.7 Carboanhydrase inhibitors

In the early 1970s, Paterson [321] and Heilmann [322] described visual field reversibility in glaucoma patients after treatment with acetazolamide. We confirmed these findings [323] and postulated a relationship with an improvement of OBF. This effect occurred particularly in young female subjects [199]. Today, we know that both the systemically applied acetazolamide as well as the locally applied dorzolamide improve OBF [324].

### 7.8 Ginkgo biloba

Oxidative stress occurs particularly in the mitochondria, where it damages the inner membrane. While vitamins do not reach this area, *G. biloba* polyphenols may protect the mitochondria. For more details, we refer to a recent review [325].

### 7.9 Other drugs

A female patient with advanced NTG and PVD took sildenafil for better vision. Indeed, sildenafil increases BF in the choroid [326], and we observed dilatation of retinal arteries and veins in healthy subjects after intake [327]. Whether a long-term treatment with a phosphodiesterase-5 inhibitor is helpful or counterproductive in PVD subjects is unclear as of now.

In *ex vivo* studies, dipyridamole dilated ciliary arteries, and pre-incubation with dipyridamole reduced contractions to ET-1 [328]. In clinical short-term studies, dipyridamole increased BF velocities in the extraocular vessels [329]. However, long-term studies are not available so far.

## 8 Conclusions

Although PVD syndrome is quite prevalent and mostly benign, it can be accompanied by bothering symptoms and may contribute to the occurrence and progression of potentially serious diseases such as NTG. Despite the emphasised importance, we are still in the infant stage of the research in this field. Taking into consideration the prevalence of PVD in the population, the authors strongly recommend the following steps to be undertaken in order to effectively promote the field:

• While in the past nailfold capillaroscopy was considered to be the gold standard for the diagnosis of PVD, we may need to redefine it. The proposed minimally invasive biomarker panels besides imaging by nailfold capillary microscopy with cold provocation are the dynamic retinal vessel imaging with flicker light stimulation or the pathology-specific molecular profiles in circulating leukocytes.

• The phenomenology of the syndrome needs to be further qualitatively and quantitatively characterised to enable early diagnosis, pathology prediction and targeted prevention in groups at risk. This task is of particular value from the viewpoint of ethics and positive economy of medical services.

• The underlying pathophysiology should be studied in more detail. Concretely, the role of the autonomic nervous system, vascular endothelial cells or even that of the mitochondria needs to be clarified in order to develop highly sensitive diagnostic tools and to promote therapy approaches tailored to the person.

• The functional link between vascular dysregulation and the spectrum of related and downstream diseases needs to be established.

• The present treatment is of limited value and not yet based on well-controlled studies. Once the role of the syndrome is established and accepted by the scientific community, innovative diagnostic tools might be created and the pharmaceutical industry can get motivated to develop novel drug targets and more efficient treatment approaches.

## Competing interests

The authors declare that they have no competing interests.

## Authors’ contributions

JF created the concept of the review, drafted the article and coordinated the writing. KK and AJF contributed to the drafting, writing and reviewing of the manuscript. All authors read and approved the final manuscript.

## Authors’ information

JF has worked in the field of glaucoma and microcirculation for decades. For more details see http://www.glaucomaresearch.ch. KK is an ophthalmologist and a glaucoma fellow with special interest in ocular blood flow. AJF is a cardiologist with special interest in vascular endothelial function.
